# Reporting Quality of Systematic Reviews/Meta-Analyses of Acupuncture

**DOI:** 10.1371/journal.pone.0113172

**Published:** 2014-11-14

**Authors:** Yali Liu, Rui Zhang, Jiao Huang, Xu Zhao, Danlu Liu, Wanting Sun, Yuefen Mai, Peng Zhang, Yajun Wang, Hua Cao, Ke hu Yang

**Affiliations:** 1 Evidence-Based Medicine Center, School of Basic Medical Sciences, Lanzhou University, Lanzhou, China; 2 Key Laboratory of Clinical Translational Research and Evidence-Based Medicine of Gansu Province, Lanzhou, China; 3 The First Clinical Medical College of Lanzhou University, Lanzhou, China; 4 The Second Clinical Medical College of Lanzhou University, Lanzhou, China; 5 Department of Cardiology, Qilu Hospital of Shandong University, Ji'nan, Shandong Province, China; 6 Acupuncture and Massage College, Gansu University of Traditional Chinese Medicine, Lanzhou, China; 7 Department of Neurology, Gansu Provincial Hospital of Traditional Chinese Medicine, Lanzhou, China; McGill University, Canada

## Abstract

**Background:**

The QUOROM and PRISMA statements were published in 1999 and 2009, respectively, to improve the consistency of reporting systematic reviews (SRs)/meta-analyses (MAs) of clinical trials. However, not all SRs/MAs adhere completely to these important standards. In particular, it is not clear how well SRs/MAs of acupuncture studies adhere to reporting standards and which reporting criteria are generally ignored in these analyses.

**Objectives:**

To evaluate reporting quality in SRs/MAs of acupuncture studies.

**Methods:**

We performed a literature search for studies published prior to 2014 using the following public archives: PubMed, EMBASE, Web of Science, the Cochrane Database of Systematic Reviews (CDSR), the Chinese Biomedical Literature Database (CBM), the Traditional Chinese Medicine (TCM) database, the Chinese Journal Full-text Database (CJFD), the Chinese Scientific Journal Full-text Database (CSJD), and the Wanfang database. Data were extracted into pre-prepared Excel data-extraction forms. Reporting quality was assessed based on the PRISMA checklist (27 items).

**Results:**

Of 476 appropriate SRs/MAs identified in our search, 203, 227, and 46 were published in Chinese journals, international journals, and the Cochrane Database, respectively. In 476 SRs/MAs, only 3 reported the information completely. By contrast, approximately 4.93% (1/203), 8.81% (2/227) and 0.00% (0/46) SRs/Mas reported less than 10 items in Chinese journals, international journals and CDSR, respectively. In general, the least frequently reported items (reported≤50%) in SRs/MAs were “protocol and registration”, “risk of bias across studies”, and “additional analyses” in both methods and results sections.

**Conclusions:**

SRs/MAs of acupuncture studies have not comprehensively reported information recommended in the PRISMA statement. Our study underscores that, in addition to focusing on careful study design and performance, attention should be paid to comprehensive reporting standards in SRs/MAs on acupuncture studies.

## Introduction

Systematic reviews (SRs) and meta-analyses (MAs) summarize large amounts of evidence and are a valuable tool for keeping clinicians up to date within their specialty [1], [2]. As with all research, however, the value of SRs/MAs depends on how the analyses are performed, the actual findings, and the clarity of reporting [3]. If key information is reported poorly, the potential usefulness of the SRs/MAs is diminished.

Since 1987, numerous researchers have recognized the need to evaluate the quality of these types of reviews. For example, in 1987 Sacks and colleagues [4] evaluated reporting in SRs/MAs and found that it was inadequate. The Consolidated Standards of Reporting Trials (CONSORT) Group subsequently developed the Quality of Reporting of Meta-Analyses (QUOROM) statement to address suboptimal MA reporting. Ten years later, an updated QUOROM statement—entitled Preferred Reporting Items for Systematic Reviews and Meta-Analyses (PRISMA) statement—was developed and published [3]. The PRISMA statement consists of a checklist of 27 study reporting items such as title, abstract, methods, results, discussion, and funding sources. The checklist is intended to guide authors of SRs/MAs to improve the consistency and quality of reporting.

Acupuncture, a traditional medicine technique, has been widely used in clinical practice for thousands of years in China and many western countries. The number of published SRs/MAs of acupuncture studies has increased considerably in recent years. As the transparency and completeness of SRs/MAs in many fields is still not optimal [5]–[8], we examined how well SR/MA reporting standards have been followed in the field of acupuncture and compared adherence to these standards in acupuncture SRs/MAs published in three different types of journals/databases.

## Methods

The protocol for this study was written in Chinese and has not been published. The study was not a classical systematic review, but we tried to report it according to PRISMA Checklist [3] (Text S1).

### Inclusion/exclusion criteria

We included all SRs or MAs of acupuncture published in Chinese journals, international journals, and the Cochrane Database of Systematic Reviews (CDSR) prior to 2014. The experimental group of SRs/MAs of acupuncture studies was also compared with a control group of SRs/MAs of studies of other interventions, such as herbal medicine, massage and western medicine. Participants: human in any conditions, not animal; Intervention: acupuncture; Comparisons: sham acupuncture or other interventions, such as herbal medicine, massage, western medicine, etc; Outcomes: no limitations; Study design: SRs/MAs. We excluded SRs/MAs that focused primarily on traditional Chinese medicine (TCM) other than acupuncture (e.g., herbal medicine, massage).

### Search strategy

We comprehensively and systematically searched the following literature archives for SRs/MAs published prior to 2014: CDSR, PubMed, EMBASE, Web of Science, Chinese Biomedical Literature Database (CBM), the TCM database, Chinese Journal Full-text Database (CJFD), Chinese Scientific Journal Full-text Database (CSJD), and the Wanfang database. Databases were searched three times: on March 24, 2011 for all entries submitted prior to March 2011, on June 12, 2012 for all entries submitted prior to January 2012 and on January 11, 2014 for all entries submitted prior to 2014. The search terms “acupuncture”, “needling”, “ear acupuncture”, “electroacupuncture”, “electro-acupuncture”, “acupuncture points”, “acupressure”, “moxibustion”, and “acupoint” were used with the terms “systematic review” or “meta-analysis”. The search strategy is presented in Checklist S1.

### Screening

The titles and abstracts of the studies were independently screened by at least two reviewers (Jiao Huang, Xu Zhao, or Rui Zhang) based on inclusion and exclusion criteria, and the full text of potentially suitable articles was retrieved for further assessment (Text S2).

### Data extraction and analysis

Data were extracted independently by at least two reviewers (Rui Zhang, Jiao Huang, Xu Zhao, or Danlu Liu) in accordance with the PRISMA checklist and the assessment checklist for SRs/MAs of acupuncture studies developed for this study. Inconsistencies were subsequently resolved by discussion between the two reviewers or final decisions were made by the third principal investigator (Yali Liu). Data input utilized a standardized form and was done by trained data extractors (Wanting Sun, Pen Zhang, and Hua Cao). The form consisted of a general characteristics section (title, first author, funding source, study design, disease(s) examined, diagnostic criteria, intervention, and outcome) and a 27-item PRISMA information section (including title, abstract, introduction, method, results, discussion, and funding). Each item was assessed as “yes” if it was described in the paper or “no” if it was not (Text S3). Data were summarized with descriptive statistical analysis. For continuous data, the means ± SD was provided and one-way ANOVA was used. Data that followed a normal distribution were compared using the LSD- *t* test. Dichotomous data were summarized with descriptive statistical analysis (frequency, percentage). Pearson's *χ* ^2^ test and/or Fisher's exact test were used to assess differences in reporting among groups. *P* values less than 0.05 were considered significant. All statistical analyses were performed using Microsoft Excel (version 2007) and SPSS (version 13.0) software.

## Results

### Search

**Figure 1:**
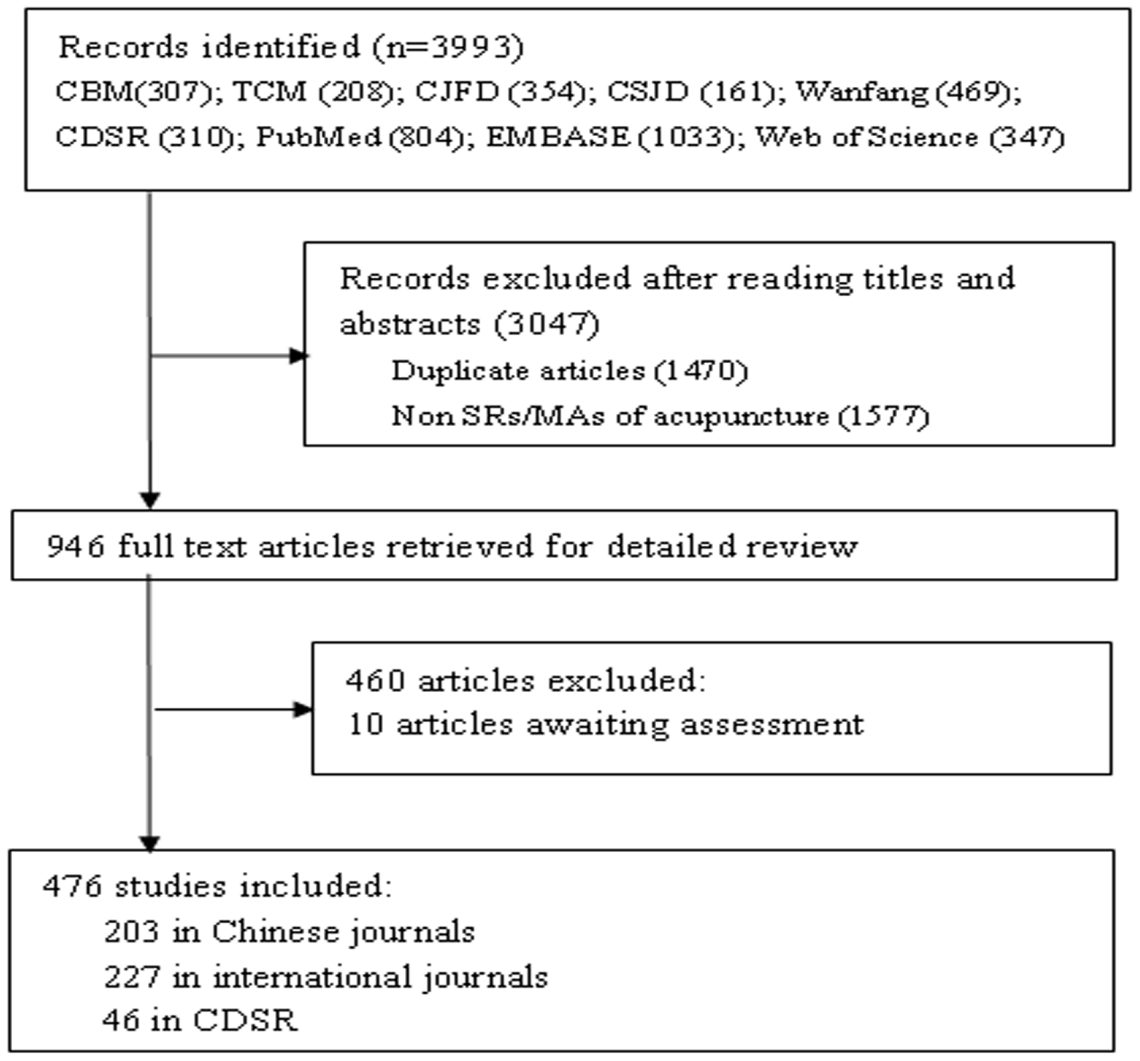
Flow chart of articles identified, included and excluded.

Our initial literature search identified 3993 potential SRs/MAs of acupuncture-related studies. After closer examination, 476 were chosen for inclusion in our analysis (Text S4). Of these, 203, 227, and 46 were published in Chinese journals, international journals, and CDSR, respectively. A flow chart of the literature search is shown in Figure 1.

### General characteristics

**Figure 2:**
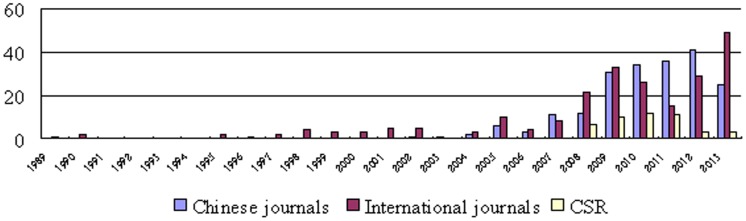
The number of included SRs MAs on acupuncture.

General characteristics of the SRs/MAs analyzed are summarized in Table 1. The earliest acupuncture SRs/MAs in Chinese journals and international journals were published in 2002 and 1989, respectively. The number of acupuncture SRs/MAs in Chinese and international journals increased sharply after 2005, whereas the majority of acupuncture SRs/MAs in CDSR were published between 2008 and 2013 (Figure 2).

**Table 1:** Characteristics of included studies.

Category	Characteristic	Chinese journals n = 203	International journals n = 227	CDSRs n = 46
Title	Systematic review	123 (122+1*)	167 (124+43*)	NA
	Meta analyses	66 (65+1*)	78 (35+43*)	NA
Author	The first author	203 (China)	62(China), 58(Korea), 39(England), 20(America)	16(China), 10(England), 9(Australia)
Funding source	Number of funded SRs/MAs	110 (54.19%)	110(48.46%)	40(86.96%)
Trial types	RCTs	193 (95.07%)	215(94.71%)	46 (100.0%)
Diseases	The first three	Nervous system 45 (22.17%), Musculoskeletal system 40 (19.70%), Mental illness 32 (15.76%)	Nervous system 57 (25.11%), Musculoskeletal system 42 (18.50%), Mental illness 27(11.89%)	Musculoskeletal system 8 (17.39%), Nervous system 8 (17.39%), Mental illness 5 (10.87%)
Diagnostic criteria	Western medicine (diseases)	74(36.45%)	80(35.24%)	19(41.30%)
	Traditional medicine	44(21.67%)	6(2.64%)	0(0.00%)
Intervention		203(100.00%)	227 (100.00%)	46 (100%)
Outcome	Including adverse effect	54 (26.60%)	103 (45.37%)	33 (71.74%)
	Including quality of life	25 (12.31%)	45(19.82%)	23 (50.00%)

* Reported both “systematic review” and “meta-analysis”.

Acupuncture SRs/MAs in Chinese journals were conducted entirely by Chinese authors, whereas those published in international journals tended to be multinational collaborations, with Chinese first authors being most prevalent (27.31%, 62/227). Chinese authors were also most prevalent first authors in CDSR studies (34.78%, 16/46). The percentage of published acupuncture SRs/MAs in Chinese journals, international journals, and the CDSR that reported at least one funding source was 54.19% (110/203), 50.66% (115/227), and 89.13% (41/46), respectively, and the maximum number of funding sources reported was 5, 3, and 7, respectively.

The majority of SRs/MAs (95.37%, 454/476) included at least one randomized controlled trial (RCT). Nervous system diseases, musculoskeletal system diseases, and mental illness were most frequently examined (23.11, 18.91 and 13.44%, respectively). Approximately 38% (180/476) of the SRs/MAs reported western diseases or TCM syndromes in their diagnostic criteria. All acupuncture SRs/MAs examined described the interventions in detail. 39.92% (190/476) and 19.54% (93/476) SRs/MAs included adverse events and quality-of-life in the outcome which were reported as primary and/or secondary outcomes.

### PRISMA information reporting

Comparison of PRISMA reporting among the three types of journals/databases (Table 2)

**Table 2:** Reporting of checklists for PRISMA statement.

Category	Item		Total n = 476	Chinese journals n = 203	International journals n = 227	CDSRs n = 46	*P* value
Title	1	Title	390(90.70%)@	188(92.61%)	202(88.99%)*	NA	NA
Abstract	2	Structured summary	446(93.70%)	199(98.03%)	201(88.55%)*	46(100.00%)#	0.000
Introduction	3	Rationale	380(79.83%)	152(74.88%)	182(80.18%)	46(100.00%)*#	0.001
	4	Objective	430(90.34%)	160(78.82%)	225(99.12%)*	45(97.83%)*	0.000
Methods	5	Protocol and registration	60(12.61%)	0(0.00%)	18(7.93%)	42(91.30%)*#	0.000
	6	Eligibility criteria	463(97.27%)	196(96.55%)	222(97.80%)	45(97.83%)	0.710
	7	Information sources	440(92.44%)	169(83.25%)	225(99.12%)*	46(100.00%)	0.000
	8	Search	282(59.24%)	87(42.86%)	156(68.72%)*	39(84.78%)	0.000
	9	Study selection	342(71.85%)	116(57.14%)	182(80.18%)*	44(95.65%)*	0.000
	10	Data collection process	411(86.34%)	159(78.33%)	207(91.19%)*	45(97.83%)	0.000
	11	Data items	270(56.72%)	59(29.06%)	170(74.89%)*	41(89.13%)*	0.000
	12	Risk of bias in individual studies	384(80.67%)	159(78.33%)	182(80.18%)	43(93.48%)	0.061
	13	Summary measures	387(81.30%)	182(89.66%)	164(72.25%)*	41(89.13%)	0.000
	14	Synthesis of results	402(84.45%)	191(94.09%)	167(73.57%)*	44(95.65%)#	0.000
	15	Risk of bias across studies	155(32.56%)	82(40.39%)	50(22.03%)*	23(50.00%)*#	0.000
	16	Additional analyses	191(40.13%)	77(37.93%)	76(33.48%)	38(82.61%)*#	0.000
Results	17	Study selection	430(90.34%)	188(92.61%)	197(86.78%)	45(97.83%)	0.024
	18	Study characteristics	431(90.55%)	176(86.70%)	209(92.07%)	46(100.00%)*#	0.012
	19	Risk of bias within studies	387(81.30%)	155(76.35%)	189(83.26%)	43(93.48%)*	0.016
	20	Results of individual studies	411(86.34%)	196(96.55%)	172(75.77%)*	43(93.48%)#	0.000
	21	Synthesis of results	389(81.72%)	192(94.58%)	156(68.72%)*	41(89.13%)#	0.000
	22	Risk of bias across studies	176(36.97%)	91(44.83%)	66(29.07%)*	19(41.30%)	0.003
	23	Additional analysis	165(34.66%)	49(24.14%)	75(33.04%)	41(89.13%)*#	0.000
Discussion	24	Summary of evidence	432(90.76%)	170(83.74%)	216(95.15%)*	46(100.00%)*	0.000
	25	Limitations	455(95.59%)	188(92.61%)	222(97.80%)	45(97.83%)	0.024
	26	Conclusions	464(97.48%)	193(95.07%)	226(99.56%)*	45(97.83%)	0.012
Funding	27	Funding	324(68.07%)	104(51.23%)	179(78.85%)*	41(89.13%)*	0.000

@ n = 430;

* # : there were statistical differences compared with Chinese journals and international journals, respectively.

Among 476 SRs/MAs, only 3 reported the information completely. By contrast, approximately 4.93% (1/203), 8.81% (2/227) and 0.00% (0/46) SRs/MAs reported less than 10 items on the checklist in Chinese journals, international journals, and CDSR, respectively. In general, the least frequently reported items (reported≤50%) in SRs/MAs were item 5 (“protocol and registration”), 15 and 22 (“risk of bias across studies”), and 16 and 23 (“additional analyses”). The remaining items on the checklist were adequately reported (i.e >90%), with the items listed in Table 2 being especially well reported.

Comparison of PRISMA reporting before and after release of the PRISMA statement (Table 3)

**Table 3:** The comparison for Reporting of checklists for SRs/MAs on PRISMA statement.

Category	Item		≦2009 year n = 186	>2009 year n = 290	*P* value	SCI n = 204	Non-SCI n = 272	*P* value
Title	1	Title	143(84.62%$)	247(95.00%#)*	0.022	145(92.36%&)	245(90.07%)*	0.000
Abstract	2	Structured summary	171(91.94%)	275(94.83%)	0.205	188(92.16%)	258(94.85%)	0.231
Introduction	3	Rationale	113(60.75%)	268(92.41%)*	0.000	172(84.31%)	208(76.47%)*	0.035
	4	Objective	185(99.46%)	246(84.83%)*	0.000	201(98.53%)	229(84.19%)*	0.000
Methods	5	Protocol and registration	24(12.90%)	36(12.41%)	0.875	56(27.45%)	4(1.47%)*	0.000
	6	Eligibility criteria	184(98.92%)	279(96.54%)	0.076	199(97.55%)	265(97.43%)	0.933
	7	Information sources	179(96.24%)	261(90.00%)*	0.012	202(99.02%)	238(87.50%)*	0.000
	8	Search	110(59.14%)	172(59.31%)	0.971	160(78.43%)	122(44.85%)*	0.000
	9	Study selection	124(66.67%)	224(75.68%)*	0.011	173(84.80%)	169(62.13%)*	0.000
	10	Data collection process	165(88.71%)	245(84.48%)	0.193	187(91.67%)	224(82.35%)*	0.003
	11	Data items	98(52.69%)	171(58.97%)	0.178	153(75.00%)	117(43.01%)*	0.000
	12	Risk of bias in individual studies	143(76.88%)	240(82.76%)	0.115	172(84.31%)	212(77.94%)	0.081
	13	Summary measures	141(75.81%)	246(84.83%)*	0.014	160(78.43%)	227(83.46%)	0.164
	14	Synthesis of results	147(79.03%)	255(87.93%)*	0.009	162(79.41%)	240(88.24%)*	0.009
	15	Risk of bias across studies	39(20.97%)	118(40.69%)*	0.000	66(32.35%)	90(33.09%)	0.866
	16	Additional analyses	66(35.48%)	125(43.10%)	0.098	97(47.55%)	95(34.93%)*	0.005
Results	17	Study selection	162(87.10%)	268(92.41%)	0.055	183(89.71%)	247(90.81%)	0.687
	18	Study characteristics	165(88.71%)	266(91.72%)	0.273	193(94.61%)	238(87.50%)*	0.009
	19	Risk of bias within studies	140(75.27%)	247(85.17%)*	0.007	176(86.27%)	212(77.94%)*	0.020
	20	Results of individual studies	139(74.73%)	272(93.79%)*	0.000	169(82.84%)	242(88.97%)	0.054
	21	Synthesis of results	134(72.04%)	255(87.93%)*	0.000	154(75.49%)	235(86.40%)*	0.002
	22	Risk of bias across studies	49(26.34%)	129(44.48%)*	0.000	69(33.82%)	108(39.71%)	0.189
	23	Additional analysis	57(30.65%)	107(36.90%)	0.161	94(46.08%)	71(26.10%)*	0.000
Discussion	24	Summary of evidence	182(97.85%)	249(85.86%)*	0.000	193(94.61%)	239(87.87%)*	0.012
	25	Limitations	183(98.39%)	272(93.79%)*	0.017	198(97.06%)	257(94.49%)	0.176
	26	Conclusions	185(99.46%)	279(96.21%)*	0.033	202(99.02%)	262 (96.32%)	0.063
Funding	27	Funding	129(69.35%)	200(68.97%)	0.929	170(83.33%)	154(56.62%)*	0.000

$ n = 169,

# n = 260,

& n = 157.

*: there were statistical differences compared with non-SCI journals/>2009 y and SCI journals/2009 y, respectively.

We found no statistical difference (*P*>0.05) for item 2 (“structured summary”), 5 (“protocol and registration”), 6 (“eligibility criteria”), 8 (“search”), 10 (“data collection process”), 11 (“data items”), 12 (“risk of bias in individual studies”), 16 (“additional analyses”), 17 (“study selection”), 18 (“study characteristics”), 23 (“additional analysis”) and 27 (“funding”) between SRs/MAs published prior to release of the PRISMA statement and those published after its release. Unfortunately, the rate of reporting of two items (“objective” and “information sources”) had decreased in 2010–2013 compared with before 2010 (*P*<0.001).

Comparison of PRISMA reporting in Science Citation Index (SCI) and non-SCI journals (Table 3)

We found that PRISMA reporting in SRs/MAs in SCI journals was more complete overall than in non-SCI journals, especially for items 4 (“objective”), 5 (“protocol and registration”), 7 (“information sources”), 8 (“search”), 9 (“study selection”), 11 (“data items”), 23 (“additional analysis”), and 27 (“funding”) (*P*<0.001).

## Discussion

Over the last decade, numerous studies have assessed the quality of reporting in SRs/MAs by their compliance with assessment instruments such as the QUOROM and PRISMA statements [9]–[12]. These studies focused predominantly on SRs/MAs covering diagnostic research and critical care. Although some quality assessment studies have looked at acupuncture SRs/MAs [13]–[15], they have focused mainly on methodological diversity in database searching, risk of bias, and heterogeneity in search strategies among CDSR. Our study compared reporting quality and PRISMA compliance in acupuncture SRs/MAs between different journal types.

We found that the five PRISMA items, namely “Protocol and registration”, “Risk of bias across studies” (both in the methods and results), and “Additional analyses” (both in the methods and results) in the methods and results, are not frequently reported, indicating that the overall quality of reporting in acupuncture SRs/MAs is far from adequate. Compared with SRs/MAs published in CDSR, those in Chinese and international journals were of inferior reporting quality. One possible explanation for the limited compliance may be that journals have failed to incorporate the PRISMA statement into their instructions to authors about submitting SRs/MAs [16]. We also found that SR/MA reporting was more complete in SCI journals than in non-SCI journals but that both require improvement in adherence to PRISMA standards.

Several studies have focused on the reporting quality of SRs/MAs covering the fields of TCM [17], [18], physical therapy [19], orthopaedics [20], and oral implantology [21] field, which showed that the reporting quality was indeed poor. Although differences exist between these results and those we repot here, the reporting of major items in the PRISMA statement was similar to what we found in our present study. Additional, Fleming PS et al. [22] found that the quality of reporting was considerably better in reviews published in CDSR (*P*<0.001) than in non-CDSR.

Both the QUOROM and PRISMA statements encourage the use of specific terms in the titles of SRs/MAs, which help to identify these studies. Because of the special title format requirements of the CDSR, however, SRs/MAs published in this database cannot conform to the QUOROM/PRISMA recommendation.

Unequivocal descriptions of the scientific background and rationale for using acupuncture in the treatment of both western diseases and TCM syndromes provide the reader with a better understanding of the research context and rationale of SRs/MAs. In this respect, SRs/MAs in the CDSR were more explicit in their descriptions than those in international or Chinese journals.

The importance of protocol consistency and registration of SRs/MAs to the transparency of reporting is underscored by the fact that they are considered key aspects of the “reporting guidelines for systematic review protocols” in the international prospective register of systematic reviews (PROSPERO) [23], [24]. We found that only SRs/MAs published in the CDSR provided protocol and registration details.

The PRISMA standards suggest that methodological details such as eligibility criteria, information sources, search strategies, study selection criteria, and data collection processes are necessary to judge the quality and accuracy of SRs/MAs. The majority of the SRs/MAs published in the CDSR adequately reported these items, whereas those published in Chinese and international journals did not. Eligibility criteria are an aspect of the PICOS criteria (participants, interventions, comparisons, outcomes, and study design) central to the PRISMA approach. We propose that it is equally important that search strategies be uniformly reported. Many international journals require information about search strategies in at least one database, and the flexibility of the CDSR layout allows reporting of search strategies for multiple databases. Chinese journals, however, rarely request search strategy information. There is also considerable need for more consistency in the databases obtain acupuncture studies. We propose that, AcuBriefs (www.acubriefs.com), AcuBase (www.acubase.fr), Acudoc2 RCT (www.acubriefs.com/), and the TCM database are the most systematic and comprehensive sources for acupuncture information. Chinese RCTs make up the highest proportion of primary studies included in acupuncture SRs/MAs. If methods for sequence generation, allocation concealment, and study blindness are not adequately described, low-quality studies [25] may mislead reviewers.

We found that there is also considerable inconsistency in reporting of study selection criteria. For example, many primary studies on acupuncture report a random allocation design but are not specific enough for the reader to determine if they are actual RCTs. We propose that these uncertainties should be clarified by contacting the primary authors to determine the appropriateness of including the studies in the SRs/MAs. Because it has been suggested that only 6.8% of acupuncture efficacy studies published in Chinese journals are based on actual RCTs [25], we strongly propose that authors of SRs/MAs verify this information prior to inclusion of studies.

Acupuncture is considered an alternative or complementary treatment to western medical interventions such as drugs and surgery, and it can be considered a separate specialty. Thus, SRs/MAs on acupuncture require not only compliance with general PRISMA reporting standards but also accurate reporting of acupuncture information. As a result, it is necessary to develop an extension of the PRISMA statement for acupuncture.

There are several limitations to our study. First, our analyses were limited to acupuncture-specific SRs/MAs and therefore may not be applicable to SRs/MAs in other fields. Second, our assessment process was not blinded, and therefore the outcomes may be influenced by publication date and other factors. Third, our assessment criteria (yes or no) did not allow partial information to be used. Fourth, our study focused primarily on acupuncture rather than other TCM. We failed to distinguish acupuncture from herbal medicine massage, or western medicine because individual SRs/MAs we included in our analysis often contained several control groups rather than one group.

In summary, SRs/MAs of acupuncture studies have not comprehensively reported the information recommended in the PRISMA statement. Our study underscores that, in addition to focusing on careful study design and performance, attention should be paid to comprehensive reporting standards when publishing SRs/MAs of acupuncture studies.

## Supporting Information

Checklist S1 **PRISMA Checklist.**
(DOC)

Text S1 **The English and Chinese databases search strategy.**
(DOC)

Text S2 **Inclusion Exclusion Section.**
(DOC)

Text S3 **Definitions of reporting items.**
(DOC)

Text S4 **476 SRs/MAs of acupuncture.**
(DOC)

## References

[1] Oxman AD, Cook DJ, Guyatt GH (1994) Users' guides to the medical literature. VI. How to use an overview. 238 Evidence-Based Medicine Working Group. JAMA 272: 1367–1371. 7933399 10.1001/jama.272.17.1367

[2] Swingler GH, Volmink J, Ioannidis JP (2003) Number of published systematic 239 reviews and global burden of disease: database analysis. BMJ 327: 1083–1084. 14604930 10.1136/bmj.327.7423.1083 PMC261743

[3] Moher D, Liberati A, Tetzlaff J, Altman DG, PRISMA Group (2009) Preferred reporting items for systematic reviews and meta-analyses: the PRISMA statement. BMJ 339: b2535. 19622551 10.1136/bmj.b2535 PMC2714657

[4] Sacks HS, Berrier J, Reitman D, Ancona-Berk VA, Chalmers TC (1987) Meta-analysis of randomized controlled trials. New Engl J Med 316: 450–455. 3807986 10.1056/NEJM198702193160806

[5] Moher D, Tetzlaff J, Tricco AC, Sampson M, Altman DG (2007) Epidemiology and reporting characteristics of systematic reviews. PLoS Med 4: e78 [PMID: 17388659]. 17388659 10.1371/journal.pmed.0040078 PMC1831728

[6] Kelly KD, Travers A, Dorgan M, Slater L, Rowe BH (2001) Evaluating the quality of systematic reviews in the emergency medicine literature. Ann Emerg Med 38: 518–526. 11679863 10.1067/mem.2001.115881

[7] Richards D (2004) The quality of systematic reviews in dentistry. Evid Based Dent 5: 17. 15238972 10.1038/sj.ebd.6400242

[8] Delaney A, Bagshaw SM, Ferland A, Manns B, Laupland KB, Doig CJ (2005) A systematic evaluation of the quality of meta-analyses in the critical care literature. Crit Care 9: R575–582. 16277721 10.1186/cc3803 PMC1297628

[9] Willis BH, Quigley M (2011) The assessment of the quality of reporting of meta-analyses in diagnostic research: a systematic review. BMC Med Res Methodol 11: 163. 22151233 10.1186/1471-2288-11-163 PMC3258221

[10] Delaney A, Bagshaw SM, Ferland A, Manns B, Laupland KB, Doig CJ (2005) A systematic evaluation of the quality of meta-analyses in the critical care literature. Crit Care 5: R575–82 [PMID: 16277721]. 10.1186/cc3803 PMC1297628 16277721

[11] Moher D, Cook DJ, Eastwood S, Olkin I, Rennie D, et al (1999) Improving the quality of reports of meta-analyses of randomised controlled trials: the QUOROM statement. Quality of Reporting of Meta-analyses. Lancet 354: 1896–1900. 10584742 10.1016/s0140-6736(99)04149-5

[12] Delaney A, Bagshaw SM, Ferland A, Manns B, Laupland KB, et al (2005) A systematic evaluation of the quality of meta-analyses in the critical care literature. Crit Care 9: 575–582. 10.1186/cc3803 PMC1297628 16277721

[13] Sood A, Sood R, Bauer BA, Ebbert JO (2005) Cochrane systematic reviews in acupuncture: methodological diversity in database searching. J Altern Complement Med 11 4: 719–722. 16131298 10.1089/acm.2005.11.719

[14] Liu Y, Yang S, Dai J, Xu Y, Zhang R, Jiang H, Yan X, Yang K (2011) Risk of Bias Tool in Systematic Reviews/Meta-Analyses of Acupuncture in Chinese Journals. PLoS One 6 12: e28130. 22174772 10.1371/journal.pone.0028130 PMC3235108

[15] Lui S, Smith EJ, Terplan M (2010) Heterogeneity in search strategies among Cochrane acupuncture reviews: is there room for improvement? Acupunct Med 28 3: 149–153. 20615852 10.1136/aim.2010.002444

[16] Tao KM, Li XQ, Zhou QH, Moher D, Ling CQ, Yu WF (2011) From QUOROM to PRISMA: A Survey of High-Impact Medical Journals' Instructions to Authors and a Review of Systematic Reviews in Anesthesia. PLoS One 6 11: e27611. 22110690 10.1371/journal.pone.0027611 PMC3217994

[17] Ma B, Guo JW, Qi GQ, Li H, Peng J, Zhang Y, et al (2011) Epidemiology, Quality and Reporting Characteristics of Systematic Reviews of Traditional Chinese Medicine Interventions Published in Chinese Journals. Plos One 6 5: e20185. 21633698 10.1371/journal.pone.0020185 PMC3102106

[18] Ma B, Qi GQ, Lin XT, Wang T, Chen ZM, Yang KH (2012) Epidemiology, quality, and reporting characteristics of systematic reviews of acupuncture interventions published in Chinese journals[J]. J Altern Complement Med 18 9: 813–817. 22924413 10.1089/acm.2011.0274

[19] Padula RS, Pires RS, Alouche SR, Chiavegato LD, Lopes AD, Costa LO (2012) Analysis of reporting of systematic reviews in physical therapy published in Portuguese. Rev Bras Fisioter 16 4: 381–8. 10.1590/s1413-35552012005000040 22858736

[20] Gagnier JJ, Kellam PJ (2013) Reporting and methodological quality of systematic reviews in the orthopaedic literature. J Bone Joint Surg Am 5, 95 11: e771–7. 10.2106/JBJS.L.00597 23780547

[21] Kiriakou J, Pandis N, Fleming PS, Madianos P, Polychronopoulou A (2013) Reporting quality of systematic review abstracts in leading oral implantology journals. J Dent 41 12: 1181–7. 24075952 10.1016/j.jdent.2013.09.006

[22] Fleming PS, Seehra J, Polychronopoulou A, Fedorowicz Z, Pandis N (2013) A PRISMA assessment of the reporting quality of systematic reviews in orthodontics. Angle Orthod 83 1: 158–163. 22720835 10.2319/032612-251.1 PMC8805538

[23] PROSPERO. international prospective register of systematic reviews. Available. http://www.crd.york.ac.uk/prospero/. 10.1186/2046-4053-1-2 PMC3348673 22587842

[24] Moher D, Shamseer L, Clarke M, et al (2011) Reporting 270 Guidelines for Systematic Review Protocols. The19 Cochrane Colloquium

[25] Wu T, Li Y, Bian Z, et al (2009) Randomized trials published in some Chinese journals: how many are randomized?. Trials 10 1: 46. 19573242 10.1186/1745-6215-10-46 PMC2716312

