# Supplementary material for: Reporting Quality of Systematic Reviews/Meta-Analyses of Acupuncture
Source: PLoS One. 2014 Nov 14;9(11):e113172. doi: 10.1371/journal.pone.0113172 (PMC4232579; doi:10.1371/journal.pone.0113172)
Supplement: Checklist S1 — PRISMA Checklist. (DOC) [file pone.0113172.s001.doc]

**Checklist S1. The PRISMA checklist**

**The PRISMA checklist**

| **Section/topic** | **#** | **Checklist item** | **Reported on page #** | **Brief description of how the criteria were handled in the meta-analysis** |
| --- | --- | --- | --- | --- |
| **TITLE** | | |  |  |
| Title | 1 | Identify the report as a systematic review, meta-analysis, or both. | - | The study based on systematic reviews/meta-analysis |
| **ABSTRACT** | | |  |  |
| Structured summary | 2 | Provide a structured summary including, as applicable: background; objectives; data sources; study eligibility criteria, participants, and interventions; study appraisal and synthesis methods; results; limitations; conclusions and implications of key findings; systematic review registration number. | √  P6.L26-45 | The abstract provided a structured summary including background, method, result and conclusion according to PLoS ONE Guidelines for Authors |
| **INTRODUCTION** | | |  |  |
| Rationale | 3 | Describe the rationale for the review in the context of what is already known. | √  P7.L57-70 |  |
| Objectives | 4 | Provide an explicit statement of questions being addressed with reference to participants, interventions, comparisons, outcomes, and study design (PICOS). | - | Our objectives were to evaluate the reporting quality in SRs/MAs of acupuncture studies. |
| **METHODS** | | |  |  |
| Protocol and registration | 5 | Indicate if a review protocol exists, if and where it can be accessed (e.g., Web address), and, if available, provide registration information including registration number. | √  P8. L75 | The protocol of this study was written in Chinese which has not published. |
| Eligibility criteria | 6 | Specify study characteristics (e.g., PICOS, length of follow-up) and report characteristics (e.g., years considered, language, publication status) used as criteria for eligibility, giving rationale. | √  P8. L76-79 | Detailed inclusion and exclusion criteria were described in the methods section. |
| Information sources | 7 | Describe all information sources (e.g., databases with dates of coverage, contact with study authors to identify additional studies) in the search and date last searched. | √  P8. L81-87 | Four English databases (CDSR, PubMed, EMBASE, Web of Scienc) and five Chinese databases (CBM, TCM database, CJFD, CSJD and Wanfang Database) were systematically searched from inception to Dec. 2011. |
| Search | 8 | Present full electronic search strategy for at least one database, including any limits used, such that it could be repeated. | √  P33 | Text S1. The search strategy |
| Study selection | 9 | State the process for selecting studies (i.e., screening, eligibility, included in systematic review, and, if applicable, included in the meta-analysis). | √  P8. L89-92 | At least two reviewers independently screened and discussed. |
| Data collection process | 10 | Describe method of data extraction from reports (e.g., piloted forms, independently, in duplicate) and any processes for obtaining and confirming data from investigators. | √  P8. L94-98 | Data about general characteristics and PRISMA checklist were independently extracted by at least two reviewers. |
| Data items | 11 | List and define all variables for which data were sought (e.g., PICOS, funding sources) and any assumptions and simplifications made. | √  P9. L100-103 | General information and the information related to PRISMA checklist were extracted and analysed. Data was summarized using descriptive statistics (frequency, percentage). |
| Risk of bias in individual studies | 12 | Describe methods used for assessing risk of bias of individual studies (including specification of whether this was done at the study or outcome level), and how this information is to be used in any data synthesis. | - | We focused on the reporting quality in SRs/MAs of acupuncture studies. |
| Summary measures | 13 | State the principal summary measures (e.g., risk ratio, difference in means). | √  P9. L105-106 | We used descriptive statistics, such as frequency, percentage. |
| Synthesis of results | 14 | Describe the methods of handling data and combining results of studies, if done, including measures of consistency (e.g., I^2^) for each meta-analysis. | - | We didn’t combine results of studies. |

Page 1 of 2

| **Section/topic** | **#** | **Checklist item** | **Reported on page #** | **Brief description of how the criteria were handled in the meta-analysis** |
| --- | --- | --- | --- | --- |
| Risk of bias across studies | 15 | Specify any assessment of risk of bias that may affect the cumulative evidence (e.g., publication bias, selective reporting within studies). | - | The item was not used in this study. |
| Additional analyses | 16 | Describe methods of additional analyses (e.g., sensitivity or subgroup analyses, meta-regression), if done, indicating which were pre-specified. | - | Additional analyses (eg. sensitivity or subgroup analyses) were not used in this study. |
| **RESULTS** | | |  |  |
| Study selection | 17 | Give numbers of studies screened, assessed for eligibility, and included in the review, with reasons for exclusions at each stage, ideally with a flow diagram. | √  P16 | Figure 1 |
| Study characteristics | 18 | For each study, present characteristics for which data were extracted (e.g., study size, PICOS, follow-up period) and provide the citations. | √  P18 | Table 1 |
| Risk of bias within studies | 19 | Present data on risk of bias of each study and, if available, any outcome level assessment (see item 12). | - | We focused on the reporting quality in SRs/MAs of acupuncture studies. |
| Results of individual studies | 20 | For all outcomes considered (benefits or harms), present, for each study: (a) simple summary data for each intervention group (b) effect estimates and confidence intervals, ideally with a forest plot. | - | We reported the result about the reporting quality in SRs/MAs of acupuncture studies.. |
| Synthesis of results | 21 | Present results of each meta-analysis done, including confidence intervals and measures of consistency. | - | Frequency and percentage were reported in this study. |
| Risk of bias across studies | 22 | Present results of any assessment of risk of bias across studies (see Item 15). | - | Risk of bias across studies was not involved in this study. |
| Additional analysis | 23 | Give results of additional analyses, if done (e.g., sensitivity or subgroup analyses, meta-regression [see Item 16]). | - | Additional analysis was not used in this study. |
| **DISCUSSION** | | |  |  |
| Summary of evidence | 24 | Summarize the main findings including the strength of evidence for each main outcome; consider their relevance to key groups (e.g., healthcare providers, users, and policy makers). | √  P11-13.  L165-212 | We discussed the main findings including the reporting quality in SRs/MAs of acupuncture studies.. |
| Limitations | 25 | Discuss limitations at study and outcome level (e.g., risk of bias), and at review-level (e.g., incomplete retrieval of identified research, reporting bias). | √  P14.  L214-217 | The three limitations were listed in the discussion section. |
| Conclusions | 26 | Provide a general interpretation of the results in the context of other evidence, and implications for future research. | √  P15.  L219-222 | SRs/MAs of acupuncture studies have not comprehensively reported those information based PRISMA statement. Our study underscores that, in addition to focusing on study design and strict performance, attention should be paid to comprehensive reporting standards when reporting SRs/MAs on acupuncture studies. |
| **FUNDING** | | |  |  |
| Funding | 27 | Describe sources of funding for the systematic review and other support (e.g., supply of data); role of funders for the systematic review. | √  P5.  L18-19 | Project supported by the National Natural Science Foundation of China (Grant No. 81373882 ). Every author has no conflicts of interest or financial ties to disclose. |

*From:*  Moher D, Liberati A, Tetzlaff J, Altman DG, The PRISMA Group (2009). Preferred Reporting Items for Systematic Reviews and Meta-Analyses: The PRISMA Statement. PLoS Med 6(6): e1000097. doi:10.1371/journal.pmed1000097

For more information, visit: **www.prisma-statement.org**.

Page 2 of 2
