# Supplementary material for: Reporting Quality of Systematic Reviews/Meta-Analyses of Acupuncture
Source: PLoS One. 2014 Nov 14;9(11):e113172. doi: 10.1371/journal.pone.0113172 (PMC4232579; doi:10.1371/journal.pone.0113172)
Supplement: Text S1 — The English and Chinese databases search strategy. (DOC) [file pone.0113172.s002.doc]

**Text S1 The English and Chinese databases search strategy.**

**PubMed Database search strategy:**

**Hyperlink address: http://www.ncbi.nlm.nih.gov/pubmed/**

#1 "Meta-Analysis" [Publication Type]

#2 Meta analysis

#3 Systematic review

#4 #1 OR #2 OR #3

#5 "Acupuncture"[Mesh]

#6 "Acupuncture Therapy"[Mesh]

#7 "Acupuncture Points"[Mesh]

#8 "Moxibustion"[Mesh]

#9 acupuncture

#10 moxibustion

#11 acupressure

#12 acupoint

#13 acupuncture point

#14 electroacupuncture

#15 auricular acupuncture

#16 ear Acupuncture

#17 #5 OR #6 OR #7 OR #8 OR #9 OR #10 OR #11 OR #12 OR #13 OR #14 OR #15 OR #16

#18 #4 AND #17

**EMBASE Database search strategy:**

**Hyperlink address: http://www.embase.com/**

#1 acupuncture'/exp

#2 moxibustion/exp OR electroacupuncture/exp

#2 acupuncture' OR moxibustion OR electroacupuncture

#4 #1 OR #2 OR #3

#5 'meta analysis'/exp

# 6 meta analysis OR systemic review

#7 #5 OR #6

#8 #4 AND #7

**Cochrane database of systematic review Database search strategy:**

**Hyperlink address:** **http://www.thecochranelibrary.com/view/0/index.html**

#1 Acupuncture Therapy/

# 2 Acupuncture

# 3 Acupuncture Analgesia OR Ear Acupuncture OR electroacupuncture OR electro-acupuncture OR Meridians OR Acupuncture Points OR Moxibustion OR acupressure OR acupoint

#4 #1 OR #2 OR #3

**Chinese Biomedicine Literature Database (CBM) search strategy:**

**Hyperlink address:** http://sinomed.imicams.ac.cn/index.jsp

#1 Systematic review/exp

#2 Systematic review

#3 Meta analysis/exp

#4 Meta analysis

#5 #1 or #2 or #3 or #4

#6 acupuncture/exp

#7 Acupuncture Therapy/exp

#8 Acupuncture Points/exp

#9 Moxibustion/exp

#10 acupuncture

#11 moxibustion

#12 acupressure

#13 acupoint

#14 acupuncture point

#15 electroacupuncture

#16 auricular acupuncture

#17 ear Acupuncture

#18 #6 or #7 or #8 or #9 or #10 or #11 or #12 or #13 or #14 or #15 or #16 or #17

#19 #5 and #18

**Traditional Chinese Medicine database (TCM database)**

**Hyperlink address:** http://cowork.cintcm.com/engine/windex.jsp

#1 Meta analysis/exp

#2 Meta analysis

#3 Systematic review

#4 #1 or #2 or #3

#5 acupuncture/exp

#6 Acupuncture Therapy/exp

#7 Acupuncture Points/exp

#8 Moxibustion/exp

#9 acupuncture

#10 moxibustion

#11 acupressure

#12 acupoint

#13 acupuncture point

#14 Electroacupuncture

#15 auricular acupuncture

#16 ear Acupuncture

#17 #5 or #6 or #7 or #8 or #9 or #10 or #11 or #12 or #13 or #14 or #15 or #16

#18 #4 and #17

**Chinese Scientific Journal Full-text Database (CSJD) search strategy**

**Hyperlink address:** http://www.cqvip.com/

#1 Systematic review

#2 Meta analysis

#3 #1 or #2

#4 acupuncture

#5 moxibustion

#6 acupressure

#7 acupoint

#8 acupuncture point

#9 Electroacupuncture

#10 auricular acupuncture

#11 ear Acupuncture

#12 #4 or #5 or #6 or #7 or #8 or #9 or #10 or #11

#13 #3 and #12

**Chinese Journal Full-text Database (CJFD) search strategy**

**Hyperlink address:** http://www.cnki.net/

#1 Systematic review

#2 Meta analysis

#3 #1 or #2

#4 acupuncture

#5 moxibustion

#6 acupressure

#7 acupoint

#8 acupuncture point

#9 Electroacupuncture

#10 auricular acupuncture

#11 ear Acupuncture

#12 #4 or #5 or #6 or #7 or #8 or #9 or #10 or #11

#13 #3 and #12

**Wanfang Database search strategy**

**Hyperlink address:** http://www.wanfangdata.com.cn/

#1 Systematic review

#2 Meta analysis

#3 #1 or #2

#4 acupuncture

#5 moxibustion

#6 acupressure

#7 acupoint

#8 acupuncture point

#9 Electroacupuncture

#10 auricular acupuncture

#11 ear Acupuncture

#12 #4 or #5 or #6 or #7 or #8 or #9 or #10 or #11

#13 #3 and #12
