# Supplementary material for: Reporting Quality of Systematic Reviews/Meta-Analyses of Acupuncture
Source: PLoS One. 2014 Nov 14;9(11):e113172. doi: 10.1371/journal.pone.0113172 (PMC4232579; doi:10.1371/journal.pone.0113172)
Supplement: Text S2 — Inclusion Exclusion Section. (DOC) [file pone.0113172.s003.doc]

**Text S2. Inclusion/Exclusion Section (Screening)**

- 1. Was the study a (Cochrane) systematic review or meta-analysis ? Y/N

*(If “unsure”: seeing full text; If “no”: EXCLUDE)*

*(If yes)* 1.2 Did the study primarily focus on acupuncture?

*(If “unsure”: seeing full text; If “no”: EXCLUDE)*

*(If yes)* 1.3 Did the study publish in Chinese journal, international journal or CDSR?

*(If “unsure”: seeing full text; If “no”: EXCLUDE)*

*(If yes)* 1.2 Was this study included? (Y/N/Unsure)

1.3 Note: ( )
