# Supplementary material for: Reporting Quality of Systematic Reviews/Meta-Analyses of Acupuncture
Source: PLoS One. 2014 Nov 14;9(11):e113172. doi: 10.1371/journal.pone.0113172 (PMC4232579; doi:10.1371/journal.pone.0113172)
Supplement: Text S3 — Definitions of reporting items. (DOC) [file pone.0113172.s004.doc]

Text S3: Definitions of reporting items

| Category | Item | | Definition of “yes” |
| --- | --- | --- | --- |
| Title | 1 | Title | It should contain a word as “systematic review”, “meta-analysis”, or both.  In this study, item 1 was not assessed in CSRs. |
| Abstract | 2 | Structured summary | There was a structured summary including, as applicable, background, objectives, method (data sources, study eligibility criteria, participants, interventions, study appraisal and synthesis methods), results, conclusions and implications of key findings |
| Introduction | 3 | Rationale | Describe the rationale of acupuncture in the context of what is already known |
|  | 4 | Objective | Provide the information of questions being addressed with reference to PICOS (participants, interventions, comparisons, outcome and study design). |
| Methods | 5 | Protocol and registration | The information of a review protocol could be found, such as web address or reference. Provide registration information including registration number.  For all CSR, it is considered “yes”. |
|  | 6 | Eligibility criteria | Provide study characteristics (such as PICOS), it is considered “yes”. |
|  | 7 | Information sources | Describe all information sources (such as databases with dates of coverage) |
|  | 8 | Search | Report electronic search strategy used for at least one database. |
|  | 9 | Study selection | Report the process for selecting studies (For instance, “Two reviewers independent selecting studies based on eligibility criteria) |
|  | 10 | Data collection process | Describe method of data extraction from reports (such as piloted forms, independently, in duplicate) and any processes for obtaining and confirming data from investigators |
|  | 11 | Data items | List all variables for which data were sought (such as PICOS, funding sources) and any assumptions and simplifications made |
|  | 12 | Risk of bias in individual studies | Describe the assessment tool and methods used for assessing risk of bias or methodology quality of individual studies, and how this information is to be used in any data synthesis |
|  | 13 | Summary measures | State the principal summary measures (such as risk ratio, difference in means). |
|  | 14 | Synthesis of results | Describe the methods of handling data and combining results of studies, if done, including measures of consistency (such as I^2^ statistic) for each meta-analysis. |
|  | 15 | Risk of bias across studies | Specify any assessment of risk of bias that may affect the cumulative evidence (such as publication bias, selective reporting within studies) |
|  | 16 | Additional analyses | Describe methods of additional analyses (such as sensitivity or subgroup analyses, meta-regression), if done, indicating which were pre-specified |
| Results | 17 | Study selection | Provide the numbers of studies screened, and included in the review, with reasons for exclusions after reading full text.  For a flow diagram, it is considered “yes”. |
|  | 18 | Study characteristics | For each study, present characteristics for which data were extracted (such as study size, PICOS, follow-up period) and provide the citations. If there was table of characteristics of included studies, it is considered “yes”. |
|  | 19 | Risk of bias within studies | Provide data on risk of bias of each study. The table or risk of bias summary should be considered “yes” |
|  | 20 | Results of individual studies | Provide the result related to every outcome. For a forest plot, it is considered “yes”. |
|  | 21 | Synthesis of results | Present results of each meta-analysis done, including confidence intervals and measures of consistency |
|  | 22 | Risk of bias across studies | Provide the information about publication bias or selective reporting within studies. Although publication bias wasn’t performed, which reason could be found, it is considered “yes”. |
|  | 23 | Additional analysis | Give results of additional analyses, if done (such as sensitivity or subgroup analyses, meta-regression). It described the reason without sensitivity or subgroup analyses. |
| Discussion | 24 | Summary of evidence | Summarise the main findings including the strength of evidence for each main outcome; consider their relevance to key groups. |
|  | 25 | Limitations | Discuss limitations in the study. For example, the effect of risk of bias for the validity; incomplete retrieval of identified research, reporting bias, et al. |
|  | 26 | Conclusions | Provide a general interpretation of the results in the context of other evidence, and implications for future research |
| Funding | 27 | Funding | Describe sources of funding for the systematic review and other support (such as supply of data) . It’s better to report the role of funders for the systematic review |
