# Supplementary material for: Reporting Quality of Systematic Reviews/Meta-Analyses of Acupuncture
Source: PLoS One. 2014 Nov 14;9(11):e113172. doi: 10.1371/journal.pone.0113172 (PMC4232579; doi:10.1371/journal.pone.0113172)
Supplement: Text S4 — 476 SRs/MAs of acupuncture. (DOC) [file pone.0113172.s005.doc]

***Text S4 Four hundred and seventy six SRs/MAs of acupuncture***

**1.** Adams D, Cheng F, Jou H, et al.The safety of pediatric acupuncture: a systematic review[J]. Pediatrics. 2011, 128(6): e1575-1587.

**2.** Ahn AC, Colbert AP, Anderson BJ, et al.Electrical properties of acupuncture points and meridians: a systematic review[J]. Bioelectromagnetics. 2008, 29(4): 245-256.

**3.** Ashenden R, Silagy CA, Lodge M, et al.A meta-analysis of the effectiveness of acupuncture in smoking cessation[J]. Drug and Alcohol Review. 1997, 16(1): 33-40.

**4.** Asher GN, Jonas DE, Coeytaux RR, et al.Auriculotherapy for pain management: a systematic review and meta-analysis of randomized controlled trials[J]. The Journal of Alternative and Complementary Medicine. 2010, 16(10): 1097-1108.

**5.** Baxter GD, Bleakley C, McDonough S.Clinical effectiveness of laser acupuncture: a systematic review[J]. Journal of acupuncture and meridian studies. 2008, 1(2): 65-82.

**6.** Bjordal J, Johnson M, Lopes-Martins R, et al.Short-term efficacy of physical interventions in osteoarthritic knee pain. A systematic review and meta-analysis of randomised placebo-controlled trials[J]. BMC Musculoskeletal Disorders. 2007, 8(1): 51.

**7.** Bower WF, Diao M, Tang J, et al.Acupuncture for nocturnal enuresis in children: a systematic review and exploration of rationale[J]. Neurourology and urodynamics. 2005, 24(3): 267-272.

**8.** Brosseau L, Judd M, Marchand S, et al.Transcutaneous electrical nerve stimulation (TENS) for the treatment of rheumatoid arthritis in the hand[J]. Cochrane Database Syst Rev. 2010, (1).

**9.** Cao H, Liu JP, Lewith GT.Traditional Chinese Medicine for treatment of fibromyalgia: a systematic review of randomized controlled trials[J]. The Journal of Alternative and Complementary Medicine. 2010, 16(4): 397-409.

**10.** Cao H, Pan X, Li H, et al.Acupuncture for treatment of insomnia: A systematic review of randomized controlled trials[J]. The Journal of Alternative and Complementary Medicine. 2009, 15(11): 1171-1186.

**11.** Casimiro L, Barnsley L, Brosseau L, et al.Acupuncture and electroacupuncture for the treatment of rheumatoid arthritis[J]. Cochrane Database Syst Rev

2010, (7).

**12.** Chang WD, Wu JH, Yang WJ, et al.Therapeutic effects of low-level laser on lateral epicondylitis from differential interventions of Chinese-Western medicine: systematic review[J]. Photomedicine and laser surgery. 2010, 28(3): 327-336.

**13.** Chao LF, Zhang AL, Liu HE, et al.The efficacy of acupoint stimulation for the management of therapy-related adverse events in patients with breast cancer: a systematic review[J]. Breast cancer research and treatment. 2009, 118(2): 255-267.

**14.** Chen HY, Shi Y, Ng CS, et al.Auricular acupuncture treatment for insomnia: a systematic review[J]. The Journal of Alternative and Complementary Medicine. 2007, 13(6): 669-676.

**15.** Chen N, Zhou M, He L, et al.Acupuncture for Bell's palsy[J]. Cochrane Database Syst Rev2010, (8).

**16.** Cheong Y, Nardo LG, Rutherford T, et al.Acupuncture and herbal medicine in in vitro fertilisation: a review of the evidence for clinical practice[J]. Human Fertility. 2010, 13(1): 3-12.

**17.** Cheong YC, Hung Yu Ng E, Ledger WL.Acupuncture and assisted conception[J]. Cochrane Database Syst Rev2009, (1).

**18.** Cheuk DK, Wong V, Chen WX.Acupuncture for autism spectrum disorders (ASD)[J]. Cochrane Database Syst Rev. 2011, (9): CD007849.

**19.** Cheuk DKL, Wong V.Acupuncture for epilepsy[J]. Cochrane Database Syst Rev2009, (4).

**20.** Cheuk DKL, Yeung J, Chung K, et al.Acupuncture for insomnia[J]. Cochrane Database Syst Rev2009, (2).

**21.** Cho S, Lee J, Thabane L, et al.Acupuncture for obesity: A systematic review and meta-analysis[J]. International Journal of Obesity. 2009, 33(2): 183-196.

**22.** Cho SH, Hwang EW.Acupuncture for primary dysmenorrhoea: a systematic review[J]. BJOG: An International Journal of Obstetrics & Gynaecology. 2010, 117(5): 509-521.

**23.** Cho SH, Kim J.Efficacy of acupuncture in management of premenstrual syndrome: a systematic review[J]. Complementary Therapies in Medicine. 2010, 18(2): 104-111.

**24.** Cho SH, Lee H, Ernst E.Acupuncture for pain relief in labour: a systematic review and meta‐analysis[J]. BJOG: An International Journal of Obstetrics & Gynaecology. 2010, 117(8): 907-920.

**25.** Cho SH, Whang WW.Acupuncture for vasomotor menopausal symptoms: a systematic review[J]. Menopause. 2009, 16(5): 1065.

**26.** Cho SH, Whang WW.Acupuncture for alcohol dependence: a systematic review[J]. Alcoholism: Clinical and Experimental Research. 2009, 33(8): 1305-1313.

**27.** Cho SH, Whang WW.Acupuncture for temporomandibular disorders: a systematic review[J]. Journal of orofacial pain. 2010, 24(2): 152.

**28.** Choi TY, Kim TH, Kang JW, et al.Moxibustion for rheumatic conditions: a systematic review and meta-analysis[J]. Clinical rheumatology. 2011, 30(7): 937-945.

**29.** Claydon LS, Chesterton LS, Barlas P, et al.Dose-specific effects of transcutaneous electrical nerve stimulation (TENS) on experimental pain: a systematic review[J]. Clin J Pain. 2011, 27(7): 635-647.

**30.** Cotchett MP, Landorf KB, Munteanu SE, et al.Effectiveness of dry needling and injections of myofascial trigger points associated with plantar heel pain: a systematic review[J]. J Foot Ankle Res. 2010, 318.

**31.** Coyle M, Smith C, Peat B.Cephalic version by moxibustion for breech presentation[J]. Cochrane Database Syst Rev. 2010, 2(11).

**32.** Cui Y, Wang Y, Liu Z.Acupuncture for restless legs syndrome[J]. Cochrane Database Syst Rev2008, (4).

**33.** Cummings TM, White AR.Needling therapies in the management of myofascial trigger point pain: a systematic review[J]. Archives of Physical Medicine and Rehabilitation. 2001, 82(7): 986-992.

**34.** Davis MA, Kononowech RW, Rolin SA, et al.Acupuncture for tension-type headache: a meta-analysis of randomized, controlled trials[J]. The Journal of Pain. 2008, 9(8): 667-677.

**35.** Dos Santos S, Hill N, Morgan A, et al.Acupuncture for Treating Common Side Effects Associated With Breast Cancer Treatment: A Systematic Review[J]. Medical Acupuncture. 2010, 22(2): 81-97.

**36.** Dowswell T, Bedwell C, Lavender T, et al.Transcutaneous electrical nerve stimulation (TENS) for pain relief in labour[J]. Cochrane Database Syst Rev. 2009, (2): CD007214.

**37.** El‐Toukhy T, Sunkara S, Khairy M, et al.A systematic review and meta‐analysis of acupuncture in in vitro fertilisation[J]. BJOG: An International Journal of Obstetrics & Gynaecology. 2008, 115(10): 1203-1213.

**38.** Ernst E, White A.Life-threatening adverse reactions after acupuncture? A systematic review[J]. PAINÂ. 1997, 71(2): 123-126.

**39.** Ernst E, White AR.Acupuncture for back pain: a meta-analysis of randomized controlled trials[J]. Archives of internal medicine. 1998, 158(20): 2235.

**40.** Ernst E, White AR.Prospective studies of the safety of acupuncture: a systematic review[J]. The American journal of medicine. 2001, 110(6): 481.

**41.** Ezzo J, Berman B, Hadhazy VA, et al.Is acupuncture effective for the treatment of chronic pain? A systematic review[J]. Pain. 2000, 86(3): 217-225.

**42.** Ezzo J, Hadhazy V, Birch S, et al.Acupuncture for osteoarthritis of the knee: a systematic review[J]. Arthritis & Rheumatism. 2001, 44(4): 819-825.

**43.** Ezzo J, Richardson MA, Vickers A, et al.Acupuncture‐point stimulation for chemotherapy‐induced nausea or vomiting[J]. Cochrane Database Syst Rev2011, (3).

**44.** Fan L, Fu W, Xu N, et al.Meta-analysis of 20 clinical, randomized, controlled trials of acupuncture for depression [J]. 中国神经再生研究 (英文版). 2010, 5(24).

**45.** Flowerdew M, Gadsby J.A review of the treatment of chronic low back pain with acupuncture-like transcutaneous electrical nerve stimulation and transcutaneous electrical nerve stimulation[J]. Complementary Therapies in Medicine. 1997, 5(4): 193-201.

**46.** Fu LM, Li JT, Wu WS.Randomized controlled trials of acupuncture for neck pain: systematic review and meta-analysis[J]. The Journal of Alternative and Complementary Medicine. 2009, 15(2): 133-145.

**47.** Furlan AD, van Tulder M, Cherkin D, et al.Acupuncture and dry-needling for low back pain: an updated systematic review within the framework of the cochrane collaboration[J]. Spine. 2005, 30(8): 944-963.

**48.** Furlan AD, van Tulder MW, Cherkin D, et al.Acupuncture and dry‐needling for low back pain[J]. Cochrane Database Syst Rev2011, (2).

**49.** Galantino ML, Sowers K, Kelly M, et al.Acupuncture as an adjuvant modality with physical therapy for patients with knee osteoarthritis[J]. Medical Acupuncture. 2009, 21(3): 157-166.

**50.** Gates S, Smith L, Foxcroft D.Auricular acupuncture for cocaine dependence[J]. Cochrane Database Syst Rev. 2008, 3.

**51.** Green S, Buchbinder R, Barnsley L, et al.Acupuncture for lateral elbow pain (Cochrane Review)[J]. Cochrane Database Syst Rev2008, 1(4).

**52.** Green S, Buchbinder R, Hetrick SE.Acupuncture for shoulder pain[J]. Cochrane Database Syst Rev2008, (4).

**53.** Helmreich RJ, Shiao S, Dune LS.Meta-analysis of acustimulation effects on nausea and vomiting in pregnant women[J]. Explore (New York, NY). 2006, 2(5): 412.

**54.** Henderson H.Acupuncture: evidence for its use in chronic low back pain[J]. British journal of nursing. 2002, 11(21): 1395-1403.

**55.** Huang W, Kutner N, Bliwise DL.A systematic review of the effects of acupuncture in treating insomnia[J]. Sleep medicine reviews. 2009, 13(1): 73.

**56.** Infante AF, Olmos LG, Gamarra AG, et al.Effectiveness of acupuncture in the treatment of pain from osteoarthritis of the knee[J]. Atención Primaria. 2002, 30(10): 602-610.

**57.** Jedel E.Acupuncture in xerostomia–a systematic review[J]. Journal of oral rehabilitation. 2005, 32(6): 392-396.

**58.** Johnson M, Martinson M.Efficacy of electrical nerve stimulation for chronic musculoskeletal pain: a meta-analysis of randomized controlled trials[J]. Pain. 2007, 130(1): 157-165.

**59.** Johnston BC, da Costa BR, Devereaux PJ, et al.The use of expertise-based randomized controlled trials to assess spinal manipulation and acupuncture for low back pain: a systematic review[J]. Spine (Phila Pa 1976). 2008, 33(8): 914-918.

**60.** Khadilkar A, Milne S, Brosseau L, et al.Transcutaneous electrical nerve stimulation for the treatment of chronic low back pain: a systematic review[J]. Spine. 2005, 30(23): 2657-2666.

**61.** Khadilkar A, Odebiyi D, Brosseau L, et al.Transcutaneous electrical nerve stimulation (TENS) versus placebo for chronic low-back pain[J]. Cochrane Database Syst Rev. 2010, 4(1).

**62.** Kim JI, Choi JY, Lee H, et al.Moxibustion for hypertension: a systematic review[J]. BMC Cardiovasc Disord. 2010, 1033.

**63.** Kim KH, Lee MS, Choi SM, et al.Acupuncture for treating uremic pruritus in patients with end-stage renal disease: A systematic review[J]. Journal of pain and symptom management. 2010, 40(1): 117-125.

**64.** Kim KH, Lee MS, Won Kang K, et al.Role of acupressure in symptom management in patients with end-stage renal disease: a systematic review[J]. Journal of palliative medicine. 2010, 13(7): 885-892.

**65.** Kim M, Choi TY, Lee MS, et al.Contralateral acupuncture versus ipsilateral acupuncture in the rehabilitation of post-stroke hemiplegic patients: a systematic review[J]. BMC complementary and alternative medicine. 2010, 10(1): 41.

**66.** Kim SY, Chae Y, Lee SM, et al.The effectiveness of moxibustion: an overview during 10 years[J]. Evidence Based Complementary and AlternativeMedicine. 2009, 201175.

**67.** Kim SY, Park HJ, Lee H.Acupuncture for premenstrual syndrome: a systematic review and meta-analysis of randomised controlled trials[J]. BJOG. 2011, 118(8): 899-915.

**68.** Kjellman G, Skargren E, Oberg B.A critical analysis of randomised clinical trials on neck pain and treatment efficacy. A review of the literature[J]. Scandinavian journal of rehabilitation medicine. 1999, 31(3): 139.

**69.** Kong JC, Lee MS, Shin BC.Randomized Clinical Trials on Acupuncture in Korean Literature[J]. Evidence-Based Complementary and Alternative Medicine. 2009, 6(1): 41-48.

**70.** Kong JC, Lee MS, Shin BC, et al.Acupuncture for functional recovery after stroke: a systematic review of sham-controlled randomized clinical trials[J]. Canadian Medical Association Journal. 2010, 182(16): 1723-1729.

**71.** Kwon Y, Pittler M, Ernst E.Acupuncture for peripheral joint osteoarthritis A systematic review and meta-analysis[J]. Rheumatology. 2006, 45(11): 1331-1337.

**72.** La Touche R, Angulo-Díaz-Parreño S, de-la-Hoz JL, et al.Effectiveness of acupuncture in the treatment of temporomandibular disorders of muscular origin: a systematic review of the last decade[J]. The Journal of Alternative and Complementary Medicine. 2010, 16(1): 107-112.

**73.** La Touche R, Goddard G, De-la-Hoz JL, et al.Acupuncture in the treatment of pain in temporomandibular disorders: a systematic review and meta-analysis of randomized controlled trials[J]. The Clinical journal of pain. 2010, 26(6): 541-550.

**74.** Lam YC, Kum WF, Durairajan SSK, et al.Efficacy and safety of acupuncture for idiopathic Parkinson's disease: a systematic review[J]. The Journal of Alternative and Complementary Medicine. 2008, 14(6): 663-671.

**75.** Langhorst J, Klose P, Musial F, et al.Efficacy of acupuncture in fibromyalgia syndrome—a systematic review with a meta-analysis of controlled clinical trials[J]. Rheumatology. 2010, 49(4): 778-788.

**76.** Law SK, Li T.Acupuncture for glaucoma[J]. Cochrane Database Syst Rev2010, (9).

**77.** Lee A, Done ML.The use of nonpharmacologic techniques to prevent postoperative nausea and vomiting: a meta-analysis[J]. Anesthesia & Analgesia. 1999, 88(6): 1362-1362.

**78.** Lee A, Fan L.Stimulation of the wrist acupuncture point P6 for preventing postoperative nausea and vomiting[J]. Cochrane Database Syst Rev. 2011, 2(1).

**79.** Lee DH, Kim JI, Lee MS, et al.Moxibustion for ulcerative colitis: a systematic review and meta-analysis[J]. BMC Gastroenterol. 2010, 1036.

**80.** Lee EJ, Frazier SK.The efficacy of acupressure for symptom management: a systematic review[J]. J Pain Symptom Manage. 2011, 42(4): 589-603.

**81.** Lee H, Ernst E.Acupuncture for labor pain management: a systematic review[J]. American journal of obstetrics and gynecology. 2004, 191(5): 1573-1579.

**82.** Lee H, Ernst E.Acupuncture for GI endoscopy: a systematic review[J]. Gastrointestinal endoscopy. 2004, 60(5): 784.

**83.** Lee H, Ernst E.Acupuncture analgesia during surgery: a systematic review[J]. Pain. 2005, 114(3): 511-517.

**84.** Lee H, Kim SY, Park J, et al.Acupuncture for lowering blood pressure: systematic review and meta-analysis[J]. American journal of hypertension. 2009, 22(1): 122-128.

**85.** Lee H, Schmidt K, Ernst E.Acupuncture for the relief of cancer‐related pain—a systematic review[J]. European Journal of Pain. 2005, 9(4): 437-437.

**86.** Lee JS, Lee MS, Min K, et al.Acupressure for treating neurological disorders: a systematic review[J]. Int J Neurosci. 2011, 121(8): 409-414.

**87.** Lee M, Shin BC, Ernst E.Acupuncture for rheumatoid arthritis: a systematic review[J]. Rheumatology. 2008, 47(12): 1747-1753.

**88.** Lee M, Shin BC, Ernst E.Acupuncture for treating menopausal hot flushes: a systematic review[J]. Climacteric. 2009, 12(1): 16-25.

**89.** Lee M, Shin BC, Ernst E.Acupuncture for Alzheimer’s disease: a systematic review[J]. International journal of clinical practice. 2009, 63(6): 874-879.

**90.** Lee M, Shin BC, Ronan P, et al.Acupuncture for schizophrenia: a systematic review and meta‐analysis[J]. International journal of clinical practice. 2009, 63(11): 1622-1633.

**91.** Lee M, Shin BC, Suen L, et al.Auricular acupuncture for insomnia: a systematic review[J]. International journal of clinical practice. 2008, 62(11): 1744-1752.

**92.** Lee MS, Choi TY, Kang JW, et al.Moxibustion for treating pain: a systematic review[J]. The American Journal of Chinese Medicine. 2010, 38(05): 829-838.

**93.** Lee MS, Choi TY, Kim JI, et al.Acupuncture for treating attention deficit hyperactivity disorder: a systematic review and meta-analysis[J]. Chin J Integr Med. 2011, 17(4): 257-260.

**94.** Lee MS, Choi TY, Park JE, et al.Effects of moxibustion for constipation treatment: a systematic review of randomized controlled trials[J]. Chin Med. 2010, 528.

**95.** Lee MS, Choi TY, Park JE, et al.Moxibustion for cancer care: a systematic review and meta-analysis[J]. BMC Cancer. 2010, 10130.

**96.** Lee MS, Choi TY, Shin BC, et al.Acupuncture for Children with Autism Spectrum Disorders: A Systematic Review of Randomized Clinical Trials[J]. J Autism Dev Disord. 2011.

**97.** Lee MS, Kim KH, Choi SM, et al.Acupuncture for treating hot flashes in breast cancer patients: a systematic review[J]. Breast cancer research and treatment. 2009, 115(3): 497-503.

**98.** Lee MS, Kim KH, Shin BC, et al.Acupuncture for treating hot flushes in men with prostate cancer: a systematic review[J]. Supportive care in cancer. 2009, 17(7): 763-770.

**99.** Lee MS, Pittler MH, Shin BC, et al.Acupuncture for allergic rhinitis: a systematic review[J]. Ann Allergy Asthma Immunol. 2009, 102(4): 269-279.

**100.** Lee MS, Pittler MH, Shin BC, et al.Bee venom acupuncture for musculoskeletal pain: a review[J]. The journal of pain: official journal of the American Pain Society. 2008, 9(4): 289.

**101.** Lee MS, Shin BC, Choi TY, et al.Acupuncture for treating dry eye: a systematic review[J]. Acta Ophthalmologica. 2011, 89(2): 101-106.

**102.** Lee MS, Shin BC, Ernst E.Acupuncture for treating erectile dysfunction: a systematic review[J]. BJU international. 2009, 104(3): 366-370.

**103.** Lee MS, Shin BC, Kim JI, et al.Moxibustion for Stroke Rehabilitation Systematic Review[J]. Stroke. 2010, 41(4): 817-820.

**104.** Lee MS, Shin BC, Kong JC, et al.Effectiveness of acupuncture for Parkinson's disease: A systematic review[J]. Movement Disorders. 2008, 23(11): 1505-1515.

**105.** Lee S, MYEONG SOOLEE, Choi JY, et al.Acupuncture and heart rate variability: a systematic review[J]. Autonomic neuroscience: basic & clinical. 2010, 155(1-2): 5-13.

**106.** Leo RJ, Ligot JSA.A systematic review of randomized controlled trials of acupuncture in the treatment of depression[J]. Journal of affective disorders. 2007, 97(1): 13-22.

**107.** Li X, Hu J, Wang X, et al.Moxibustion and other acupuncture point stimulation methods to treat breech presentation: a systematic review of clinical trials[J]. BMC Chinese Medicine. 2009, 44-14.

**108.** Lim B, Manheimer E, Lao L, et al.Acupuncture for treatment of irritable bowel syndrome[J]. Cochrane Database Syst Rev2010, (11).

**109.** Linde K, Allais G, Brinkhaus B, et al.Acupuncture for migraine prophylaxis[J]. Cochrane Database Syst Rev2009, (4).

**110.** Linde K, Allais G, Brinkhaus B, et al.Acupuncture for tension‐type headache[J]. Cochrane Database Syst Rev2009, (4).

**111.** Linde K, Niemann K, Schneider A, et al.How large are the nonspecific effects of acupuncture? A meta-analysis of randomized controlled trials[J]. BMC medicine. 2010, 8(1): 75.

**112.** Linde K, Vested Madsen M, Gøtzsche PC, et al.Acupuncture Treatment for Pain: Systematic Review of Randomised Clinical Trials with Acupuncture, Placebo Acupuncture, and no Acupuncture Groups[J]. Deutsche Zeitschrift für Akupunktur. 2009, 53(2): 40-41.

**113.** Liu M, He L, Wu B, et al.Acupuncture for acute stroke[J]. Cochrane Database Syst Rev2008, (4).

**114.** Liu M, Lan L, Tang Y, et al.An acupuncture meta-analysis for optic atrophy[J].

**115.** Liu T, Shi J, Epstein DH, et al.A meta-analysis of acupuncture combined with opioid receptor agonists for treatment of opiate-withdrawal symptoms[J]. Cellular and molecular neurobiology. 2009, 29(4): 449-454.

**116.** Long AF, Xing M, Morgan K, et al.Exploring the Evidence Base for Acupuncture in the Treatment of Meniere's Syndrome-A Systematic Review[J]. Evid Based Complement Alternat Med. 2009, 2011429102.

**117.** Lu S, Zheng Z, Xue CC.Does Acupuncture Improve Quality of Life for Patients with Pain Associated with the Spine? A Systematic Review[J]. Evidence-Based Complementary and Alternative Medicine. 2010, 2011.

**118.** Manheimer E, Cheng K, Linde K, et al.Acupuncture for peripheral joint osteoarthritis[J]. Cochrane Database Syst Rev2010, (1).

**119.** Manheimer E, Linde K, Lao L, et al.Meta-analysis: acupuncture for osteoarthritis of the knee[J]. Annals of Internal Medicine. 2007, 146(12): 868.

**120.** Manheimer E, White A, Berman B, et al.Meta-analysis: acupuncture for low back pain[J]. Ann Intern Med. 2005, 142(8): 651-663.

**121.** Manheimer E, Zhang G, Udoff L, et al.Effects of acupuncture on rates of pregnancy and live birth among women undergoing in vitro fertilisation: systematic review and meta-analysis[J]. Bmj. 2008, 336(7643): 545-549.

**122.** Martin J, Donaldson A, Villarroel R, et al.Efficacy of acupuncture in asthma: systematic review and meta-analysis of published data from 11 randomised controlled trials[J]. European Respiratory Journal. 2002, 20(4): 846-852.

**123.** Martin-Sanchez E, Torralba E, Díaz-Domínguez E, et al.Efficacy of acupuncture for the treatment of fibromyalgia: systematic review and meta-analysis of randomized trials[J]. The open rheumatology journal. 2009, 325.

**124.** Mayhew E, Ernst E.Acupuncture for fibromyalgia—a systematic review of randomized clinical trials[J]. Rheumatology. 2007, 46(5): 801-804.

**125.** McCarney RW, Brinkhaus B, Lasserson TJ, et al.Acupuncture for chronic asthma[J]. Cochrane Database Syst Rev2009, (3).

**126.** Melchart D, Linde K, Fischer P, et al.Acupuncture for recurrent headaches: a systematic review of randomized controlled trials[J]. Cephalalgia. 1999, 19(9): 779-786.

**127.** Mills EJ, Wu P, Gagnier J, et al.Efficacy of acupuncture for cocaine dependence: a systematic review & meta-analysis[J]. Harm Reduction Journal. 2005, 2(1): 4.

**128.** Mukaino Y, Park J, White A, et al.The effectiveness of acupuncture for depression–a systematic review of randomised controlled trials[J]. Acupuncture in Medicine. 2005, 23(2): 70-76.

**129.** Paley CA, Johnson MI, Tashani OA, et al.Acupuncture for cancer pain in adults[J]. Cochrane Database Syst Rev2011, (1).

**130.** Park J, Hopwood V, White AR, et al.Effectiveness of acupuncture for stroke: a systematic review[J]. Journal of neurology. 2001, 248(7): 558-563.

**131.** Park J, White AR, Ernst E.Efficacy of acupuncture as a treatment for tinnitus: a systematic review[J]. Archives of Otolaryngology—Head & Neck Surgery. 2000, 126(4): 489.

**132.** PATEL M, GUTZWILLER F, PACCAUD F, et al.A meta-analysis of acupuncture for chronic pain[J]. International Journal of Epidemiology. 1989, 18(4): 900-906.

**133.** Pilkington K, Kirkwood G, Rampes H, et al.Acupuncture for anxiety and anxiety disorders–a systematic literature review[J]. Acupuncture in Medicine. 2007, 25(1-2): 1-10.

**134.** Pirotta MV, White AR.Acupuncture for pelvic and back pain in pregnancy: a systematic review[J]. American Journal of Obstetrics & Gynecology. 2008.

**135.** Posadzki P, Zhang J, Lee MS, et al.Acupuncture for chronic nonbacterial prostatitis/chronic pelvic pain syndrome: a systematic review[J]. Journal of andrology. 2011, 33(1): 15.

**136.** Proctor M, Farquhar C, Stones W, et al.Transcutaneous electrical nerve stimulation for primary dysmenorrhoea[J]. Cochrane Database Syst Rev2010, 1(1).

**137.** Rathbone J, Xia J.Acupuncture for schizophrenia[J]. Cochrane Database Syst Rev2010, (12).

**138.** Robb K, Oxberry SG, Bennett MI, et al.A cochrane systematic review of transcutaneous electrical nerve stimulation for cancer pain[J]. Journal of pain and symptom management. 2009, 37(4): 746-753.

**139.** Robb KA, Bennett MI, Johnson MI, et al.Transcutaneous electric nerve stimulation (TENS) for cancer pain in adults[J]. Cochrane Database Syst Rev2008, (3).

**140.** Roberts J, Huissoon A, Dretzke J, et al.A systematic review of the clinical effectiveness of acupuncture for allergic rhinitis[J]. BMC complementary and alternative medicine. 2008, 8(1): 13.

**141.** Rosted P.The use of acupuncture in dentistry: a systematic review[J]. Acupuncture in Medicine. 1998, 16(1): 43-48.

**142.** Schneider A, Streitberger K, Joos S.Acupuncture treatment in gastrointestinal diseases: A systematic review[J]. World Journal of Gastroenterology. 2007, 13(25): 3417.

**143.** Shen FY, Lee MS, Jung SK.Effectiveness of pharmacopuncture for asthma: a systematic review and meta-analysis[J]. Evidence-Based Complementary and Alternative Medicine. 2011, 2011.

**144.** Shin BC, Lee MS, Kong JC, et al.Acupuncture for spinal cord injury survivors in Chinese literature: A systematic review[J]. Complementary Therapies in Medicine. 2009, 17(5): 316-327.

**145.** Sim H, Shin BC, Lee MS, et al.Acupuncture for carpal tunnel syndrome: a systematic review of randomized controlled trials[J]. The Journal of Pain. 2011, 12(3): 307-314.

**146.** Smith CA, Crowther CA.Acupuncture for induction of labour[J]. Cochrane Database Syst Rev2009, (1).

**147.** Smith CA, Hay PPJ, MacPherson H.Acupuncture for depression[J]. Cochrane Database Syst Rev2010, (1).

**148.** Smith CA, Zhu X, He L, et al.Acupuncture for primary dysmenorrhoea[J]. Cochrane Database Syst Rev. 2011, (1): CD007854.

**149.** Smith Caroline A, Collins Carmel T, Crowther Caroline A, et al. Acupuncture or acupressure for pain management in labour. In: Cochrane Database of Systematic Reviews: John Wiley & Sons, Ltd, 2011.

**150.** Smith LA, Oldman AD, McQuay HJ, et al.Teasing apart quality and validity in systematic reviews: an example from acupuncture trials in chronic neck and back pain[J]. Pain. 2000, 86(1-2): 119.

**151.** Sun Y, Gan T, Dubose J, et al.Acupuncture and related techniques for postoperative pain: a systematic review of randomized controlled trials[J]. British journal of anaesthesia. 2008, 101(2): 151-160.

**152.** Sun Y, Gan TJ.Acupuncture for the management of chronic headache: a systematic review[J]. Anesthesia & Analgesia. 2008, 107(6): 2038-2047.

**153.** Suzuki M, Yokoyama Y, Yamazaki H.Research into acupuncture for respiratory disease in Japan: a systematic review[J]. Acupuncture in Medicine. 2009, 27(2): 54-60.

**154.** Sze FK, Wong E, Or KKH, et al.Does acupuncture improve motor recovery after stroke? A meta-analysis of randomized controlled trials[J]. Stroke. 2002, 33(11): 2604-2619.

**155.** Ter Riet G, Kleijnen J, Knipschild P.A meta-analysis of studies into the effect of acupuncture on addiction[J]. The British journal of general practice. 1990, 40(338): 379.

**156.** Ter Riet G, Kleunen J, Knipschild P.Acupuncture and chronic pain: a criteria-based meta-analysis[J]. Journal of clinical epidemiology. 1990, 43(11): 1191-1199.

**157.** Tough EA, White AR, Cummings TM, et al.Acupuncture and dry needling in the management of myofascial trigger point pain: a systematic review and meta-analysis of randomised controlled trials[J]. European Journal of Pain. 2009, 13(1): 3-10.

**158.** Trigkilidas D.Acupuncture therapy for chronic lower back pain: a systematic review[J]. Annals of the Royal College of Surgeons of England. 2010, 92(7): 595.

**159.** Trinh K, Graham N, Gross A, et al.Acupuncture for neck disorders[J]. Cochrane Database Syst Rev2010, (3).

**160.** Trinh K, Phillips SD, Ho E, et al.Acupuncture for the alleviation of lateral epicondyle pain: a systematic review[J]. Rheumatology. 2004, 43(9): 1085-1090.

**161.** Usichenko T, Lehmann C, Ernst E.Auricular acupuncture for postoperative pain control: a systematic review of randomised clinical trials[J]. Anaesthesia. 2008, 63(12): 1343-1348.

**162.** van den Berg I, Bosch JL, Jacobs B, et al.Effectiveness of acupuncture-type interventions versus expectant management to correct breech presentation: a systematic review[J]. Complementary Therapies in Medicine. 2008, 16(2): 92-100.

**163.** Vickers A, Goyal N, Harland R, et al.Do certain countries produce only positive results? A systematic review of controlled trials[J]. Controlled clinical trials. 1998, 19(2): 159-166.

**164.** Wang C, de Pablo P, Chen X, et al.Acupuncture for pain relief in patients with rheumatoid arthritis: a systematic review[J]. Arthritis Care & Research. 2008, 59(9): 1249-1256.

**165.** Wang H, Qi H, Wang B, et al.Is acupuncture beneficial in depression: A meta-analysis of 8 randomized controlled trials?[J]. Journal of affective disorders. 2008, 111(2-3): 125-134.

**166.** Wang T, Zhang Q, Xue X, et al.A systematic review of acupuncture and moxibustion treatment for chronic fatigue syndrome in China[J]. The American Journal of Chinese Medicine. 2008, 36(01): 1-24.

**167.** Wei ML, Liu JP, Li N, et al.Acupuncture for slowing the progression of myopia in children and adolescents[J]. Cochrane Database Syst Rev. 2011, (9): CD007842.

**168.** Weina P, Zhao H, Zhishun L, et al.Acupuncture for vascular dementia[J]. Cochrane Database Syst Rev. 2009, 2(1).

**169.** White A, Ernst E.A systematic review of randomized controlled trials of acupuncture for neck pain[J]. Rheumatology. 1999, 38(2): 143-147.

**170.** White A, Foster N, Cummings M, et al.Acupuncture treatment for chronic knee pain: a systematic review[J]. Rheumatology. 2007, 46(3): 384-390.

**171.** White A, Moody R.The effects of auricular acupuncture on smoking cessation may not depend on the point chosen–an exploratory meta-analysis[J]. Acupuncture in Medicine. 2006, 24(4): 149-156.

**172.** White A, Rampes H, Campbell J.Acupuncture and related interventions for smoking cessation[J]. Cochrane Database Syst Rev. 2011, 1(1).

**173.** White AR, Resch KL, Ernst E.A meta-analysis of acupuncture techniques for smoking cessation[J]. Tobacco control. 1999, 8(4): 393-397.

**174.** White E.Acupuncture as a treatment for Temporomandibular joint dysfunction[J]. Arch Otolaryngol Head Neck Surg. 1999, 125269-272.

**175.** White P, Lewith G, Berman B, et al.Reviews of acupuncture for chronic neck pain: pitfalls in conducting systematic reviews[J]. Rheumatology. 2002, 41(11): 1224-1231.

**176.**  Wu HM, Tang JL, Lin XP, et al.Acupuncture for stroke rehabilitation[J]. Cochrane Database Syst Rev2009, (1).

**177.** Wu P, Mills E, Moher D, et al.Acupuncture in Poststroke Rehabilitation A Systematic Review and Meta-Analysis of Randomized Trials[J]. Stroke. 2010, 41(4): e171-e179.

**178.** Xie Y, Wang L, He J, et al.Acupuncture for dysphagia in acute stroke[J]. Cochrane Database Syst Rev2008, (3).

**179.** Yamashita H, Tsukayama H, White A, et al.Systematic review of adverse events following acupuncture: the Japanese literature[J]. Complementary Therapies in Medicine. 2001, 9(2): 98.

**180.** Yang H, LIU CUNZHI, Chen X, et al.Systematic review of clinical trials of acupuncture‐related therapies for primary dysmenorrhea[J]. Acta obstetricia et gynecologica Scandinavica. 2008, 87(11): 1114-1122.

**181.** Yeung WF, Chung KF, Leung YK, et al.Traditional needle acupuncture treatment for insomnia: a systematic review of randomized controlled trials[J]. Sleep medicine. 2009, 10(7): 694-704.

**182.** Yuan J, Kerr D, Park J, et al.Treatment regimens of acupuncture for low back pain--a systematic review[J]. Complement Ther Med. 2008, 16(5): 295-304.

**183.** Zhang C, Yang A, Zhang A, et al.Ear‐acupressure for allergic rhinitis: a systematic review[J]. Clinical Otolaryngology. 2010, 35(1): 6-12.

**184.** Zhang H, Bian Z, Lin Z.Are acupoints specific for diseases? A systematic review of the randomized controlled trials with sham acupuncture controls[J]. Chin Med. 2010, 5(1).

**185.** Zhang Y, Peng W, Clarke J, et al.Acupuncture for uterine fibroids[J]. Cochrane Database Syst Rev2010, (11).

**186.** Zhang ZJ, Chen HY, Yip K, et al.The effectiveness and safety of acupuncture therapy in depressive disorders: systematic review and meta-analysis[J]. Journal of affective disorders. 2010, 124(1): 9.

**187.** Zhao L, Guo Y, Wang W, et al.Systematic review on randomized controlled clinical trials of acupuncture therapy for neurovascular headache[J]. Chin J Integr Med. 2011, 17(8): 580-586.

**188.** Zheng G, Zhao ZM, Wang Y, et al.Meta-analysis of scalp acupuncture for acute hypertensive intracerebral hemorrhage[J]. The Journal of Alternative and Complementary Medicine. 2011, 17(4): 293-299.

**189.** Zhu X, Hamilton Kindreth D, McNicol Ewan D. Acupuncture for pain in endometriosis. In: Cochrane Database of Systematic Reviews: John Wiley & Sons, Ltd, 2011.

**190.** 边兴坤, 傅立新, 左小宏, 等.针刺与药物治疗抑郁症疗效比较的系统评价[J]. 针灸临床杂志. 2011, (8): 59-62.

**191.** 蔡燕, 彭楚湘.针灸治疗糖尿病的 Meta 分析[J]. 中华中医药学刊. 2010, 11067.

**192.** 曹平, 阳仁达.针灸治疗糖尿病周围神经病变的 meta 分析[J]. 中医药导报. 2011, 17(1): 97-101.

**193.** 柴华, 黎波, 杜元灏.针灸对照西药治疗膝骨性关节炎的疗效分析[J]. 辽宁中医杂志. 2009, 36(7): 1197-1200.

**194.** 陈程, 崔海福, 严兴科, 等.针灸治疗消化性溃疡的Meta分析[J]. 世界华人消化杂志. 2011, (22): 2399-2404.

**195.** 陈蓉, 黄健玲, 贡欣.针药治疗慢性盆腔炎相关文献的系统评价[J]. 陕西中医. 2011, (10): 1389-1391+1411.

**196.** 陈文, 马维平, 俞海虹, 等.针灸治疗椎-基底动脉供血不足综合征的 Meta 分析[J]. 中国中医药信息杂志. 2009, 16(8): 107-109.

**197.** 陈元武, 杜元灏, 熊俊, 等.针灸与西药治疗前列腺增生症疗效比较的系统评价[J]. 中华中医药杂志. 2010, 25(6): 902-906.

**198.** 成泽东, 陈以国, 张涛, 等.针灸治疗慢性疲劳综合征的系统评价[J]. 中国中医药信息杂志. 2011, (9): 30-32.

**199.** 樊凌, 符文彬, 许能贵, 等.针灸治疗抑郁随机对照试验的临床文献系统评价[J]. 中国老年学杂志. 2010, 30(18): 2561-2563.

**200.** 朱晓平, 符文彬, 张光彩, 等.针灸治疗颈椎病远期疗效的系统评价[J]. 中国老年学杂志. 2011, (6): 918-921.

**201.** 范祯祯.针灸治疗单纯性肥胖症的系统评价[J]. 健康必读：下半月. 2011, (9).

**202.** 付玲, 高岑.夹脊穴治疗带状疱疹临床对照研究文献的 Meta 分析[J]. 中国中西医结合皮肤性病学杂志. 2009, 8(2): 112-117.

**203.** 高锐, 时春虎, 田金徽, 等.针灸治疗青光眼的系统评价[J]. 中国针灸. 2011, (12): 1142-1145.

**204.** 高翔, 杜元灏, 黎波, 等.针刺对照西药治疗纤维肌痛综合征疗效比较的系统评价[J]. 中国疼痛医学杂志. 2010, (2): 112-114.

**205.** 高翔, 杜元灏, 肖丽, 等.国内针刺对照西药治疗三叉神经痛疗效比较的系统评价[J]. 江苏中医药. 2010, (1): 52-54.

**206.** 高小梅, 王柏松, 宋艳艳, 等.针刺治疗偏头痛的系统评价和Meta分析[J]. 中国临床药理学与治疗学. 2011, (5): 530-537.

**207.** 龚晓明, 任凯.针灸疗法在强直性脊柱炎康复治疗中的系统评价[J]. 中国康复医学杂志. 2007, 22(6): 537-539.

**208.** 郭小溪, 金红妹, 霍丽, 等.针灸治疗老年性痴呆的 Meta-分析[J]. 中国针灸. 2008, 28(2): 140-144.

**209.** 何竞, 郑敏, 何成奇, 等.穴位刺激疗法治疗脑卒中后吞咽障碍的系统评价[J]. 中国针灸. 2009, (1): 66-71.

**210.** 何俐, 周沐科, 周东, 等.针灸治疗 Bell's 面瘫疗效的系统评价[J]. 中国循证医学杂志. 2005, 5(2): 106-109.

**211.** 黄锦军, 彭文琦, 雷龙鸣, 等.中医综合疗法治疗腰椎间盘突出症的系统评价[J]. 中医药信息. 2009, 26(5): 65-69.

**212.** 黄小冬, 傅立新, 李胜, 等.针刺加耳穴贴压治疗失眠疗效的系统评价[J]. 针灸临床杂志. 2010, (11): 56-59.

**213.** 黄叶, 杨拯, 田芸, 等.小针刀治疗腰椎间盘突出症的系统评价[J]. 时珍国医国药. 2010, 21(9): 2420-2422.

**214.** 黄叶飞, 符文彬, 吴泰相, 等. 针灸治疗围绝经期抑郁症有效性和安全性的系统评价. 中华中医药杂志, 2011,26(5):908-914

**215.** 金侣位, 朱江.针灸治疗低血压的系统评价[J]. 针灸临床杂志. 2009, 25(5): 37-41.

**216.** 兰蕾, 刘迈兰, 唐勇, 等.针灸治疗原发性痛经的疗效追踪评价[J]. 山东中医药大学学报. 2009, 33(6): 511-514.

**217.** 黎波, 柴华, 杜元灏, 等.针灸治疗痤疮临床随机对照试验疗效及安全性评价[J]. 中国针灸. 2009, (3): 247-251.

**218.** 黎波, 石磊, 杜元灏, 等.针灸治疗慢性荨麻疹随机对照临床试验的系统评价[J]. 中医杂志. 2009, (5): 432-436.

**219.** 黎波, 熊峻, 杜元灏, 等.透刺与常规针刺治疗周围性面瘫疗效系统评价[J]. 辽宁中医杂志. 2009, (8): 1381-1383.

**220.** 李慧, 梁伟雄, 郭新峰.醒脑开窍针法治疗中风的 Meta 分析[J]. 广州中医药大学学报. 2004, 21(3): 215-219.

**221.** 李林, 詹红生, 陈博, 等.针刺夹脊穴治疗腰椎间盘突出症临床随机对照试验系统评价[J]. 中华中医药学刊. 2011, (6): 1208-1212.

**222.** 李林, 詹红生, 高宁阳, 等.电针治疗腰椎间盘突出症临床随机对照试验系统评价[J]. 中华中医药杂志. 2010, 25(12): 1949-1952.

**223.** 李宁, 吴滨, 王成伟, 等.针灸治疗失眠症随机对照试验的系统评价[J]. 中国针灸. 2005, 25(1): 7-10.

**224.** 李宁, 邹军.针灸治疗中风偏瘫的荟萃分析[J]. 成都中医药大学学报. 2002, 25(2): 37-39.

**225.** 李庆云, 彭唯娜, 穆岩, 等.电针治疗腰椎间盘突出症的系统评价 [J]. 现代中西医结合杂志. 2008, 17(3): 325-330.

**226.** 李胜, 傅立新, 黄小冬, 等.针刺治疗中风后假球麻痹的文献系统评价[J]. 针灸临床杂志. 2011, 27(1): 1-6.

**227.** 李漾, 罗纯.针刺治疗紧张性头痛疗效的系统评价[J]. 中国循证医学杂志. 2005, 5(2): 117-124.

**228.** 李颖, 杜元灏, 石磊, 等.针灸疗法治疗慢性浅表性胃炎的Meta分析[J]. 针灸临床杂志. 2011, (2): 1-6.

**229.** 李颖, 熊俊, 杜元灏, 等.针刺与药物治疗慢性前列腺炎疗效比较的系统评价[J]. 辽宁中医杂志. 2010, (8): 1567-1572.

**230.** 李滋平, 闫晓燕.针刺治疗原发性失眠随机对照试验的系统性评价[J]. 针灸临床杂志. 2010, (7): 43-48.

**231.** 栗丽娜, 黎波, 熊俊, 等.针刺对照激素治疗贝尔麻痹急性期疗效比较系统评价[J]. 辽宁中医药大学学报. 2010, 12(9): 97-99.

**232.** 廖丛, 周江堡.针灸治疗脑瘫的Meta分析[J]. 中国全科医学. 2011, (11): 1229-1231.

**233.** 廖良, 韦企平.国内文献有关针刺对视神经萎缩疗效的循证医学评价[J]. 中国中医眼科杂志. 2007, 17(4): 208-210.

**234.** 林汉凌, 宋红梅, 仲卫红, 等.不同针灸方法治疗神经根型颈椎病效果的系统评价[J]. 中国组织工程研究与临床康复. 2009, 13(46):9017-9021.

**235.** 林玲.穴位埋线治疗单纯性肥胖的meta分析[J]. 按摩与康复医学. 2011, 2(22):8-10.

**236.** 林小苗, 杜元灏, 熊俊, 等.国内针刺对照西药治疗脑卒中后呃逆疗效比较的系统评价[J]. 中国康复医学杂志. 2010, (4): 353-356.

**237.** 林小苗, 黎波, 杜元灏, 等.针刺治疗单纯性肥胖症临床疗效比较的系统评价[J]. 中国针灸. 2009, (10): 856-860.

**238.** 刘金欢, 陈军, 严定芳.针灸对轻度认知功能障碍治疗效果的Meta分析[J]. 长春中医药大学学报. 2011, (4): 537-538.

**239.** 刘迈兰, 兰蕾, 梁繁荣.针灸治疗臀先露的系统评价[J]. 中国循证医学杂志. 2009, 9(8): 840-843.

**240.** 刘倩, 黎波, 杜元灏, 等.针刺远近端腧穴治疗急性腰扭伤疗效比较评价[J]. 辽宁中医杂志. 2009, (8): 1392-1394.

**241.** 刘向前, 邓晋丰, 林定坤.神经根型颈椎病针刀治疗临床文献的评价 [J]. 中国中医骨伤科杂志. 2007, 15(4): 34-37.

**242.** 刘志丹, 李海燕, 宋毅, 等.针灸治疗中风运动功能障碍随机对照临床研究文献系统评价[J]. 上海针灸杂志. 2009, 27(11): 38-42.

**243.** 卢引明, 傅立新, 牟蛟, 等.针灸治疗中风后肩手综合征的系统评价[J]. 中国循证医学杂志. 2009, 9(9): 976-978.

**244.** 罗胜, 罗思文.针灸治疗腰椎间盘突出症的系统分析[J]. 针灸临床杂志. 2005, 21(6): 10-14.

**245.** 罗顺元, 杨婧, 陈梅桂.针灸治疗类风湿关节炎的 Meta 分析[J]. 中国卫生统计. 2009, (4): 431-433.

**246.** 骆雄飞, 黎波, 杜元灏, 等.针刺配合穴位注射与肌肉注射治疗周围性面瘫随机对照试验疗效评价[J]. 天津中医药. 2009, 26(1): 27-29.

**247.** 马铁明, 白增华, 任路, 等.针刺治疗焦虑症疗效 Meta 分析[J]. 中国中医药信息杂志. 2007, 14(2): 101-103.

**248.** 穆敬平, 刘莉, 方伟, 等.针刀治疗腰椎间盘突出症的系统评价[J]. Chinese Journal of Information on TCM Jul. 2010, 17(7).

**249.** 穆敬平, 吴焕淦, 张志权, 等.针灸治疗溃疡性结肠炎的 Meta 分析[J]. 中国针灸. 2007, 27(9): 687-690.

**250.** 聂容荣, 符文彬, 熊俊, 等.针灸与西药治疗脑卒中后抑郁症疗效比较的系统评价[J]. 世界中医药. 2010, 2147-151.

**251.** 潘江, 章薇, 陈武善, 等.针刺治疗周围性面瘫(急性期)的系统评价[J]. 针灸临床杂志. 2011, (4): 60-63.

**252.** 庞勇, 吴椋冰, 刘东华.针刺治疗中风失语症疗效的系统评价[J]. 中国针灸. 2010, (7): 612-616.

**253.** 彭浩, 彭海东, 许玲, 等.针刺治疗癌痛疗效的系统综述[J]. 中西医结合学报. 2010, 8(6): 501-509.

**254.** 彭唯娜, 王漪, 刘保延, 等.针灸治疗肩周炎的系统评价[J]. 世界针灸杂志: 英文版. 2007, 17(2): 1-15.

**255.** 彭唯娜, 赵宏, 刘志顺, 等.电针治疗血管性痴呆的系统评价[J]. 中国针灸. 2004, 24(5): 297-301.

**256.** 朴慧花, 于涛, 高翔, 等.针刺对照西药治疗恶性肿瘤化疗后胃肠反应临床疗效的系统评价[J]. 时珍国医国药. 2010, 21(6): 1476-1478.

**257.** 祁营洲, 傅立新, 熊俊, 等.针刺治疗中风后痉挛性瘫痪的系统评价[J]. 中国针灸. 2009, (8): 683-688.

**258.** 宋红梅, 陈少清, 王诗忠, 等.保守疗法治疗脊髓型颈椎病效果的系统评价[J]. 中国组织工程研究与临床康复. 2009, 12(46): 9149-9152.

**259.** 孙阁, 周智梁, 赵磊.腕踝针治疗疼痛类疾病临床疗效的系统评价[J]. 河北中医. 2011, 33(11): 1715-1719.

**260.** 孙攀, 杜元灏, 熊俊, 等.针刺与牵引治疗神经根型颈椎病疗效比较的系统评价[J]. 光明中医. 2009, (10): 1824-1830.

**261.** 孙燕丽, 陈爽白, 高轶, 等.国内针刺与西药治疗抑郁症疗效比较的系统评价[J]. 中国循证医学杂志. 2008, 8(5): 340-345.

**262.** 汤志梅, 张建梅, 刘佑韧, 等.针刺治疗糖尿病性胃轻瘫的系统评价[J]. 中国热带医学. 2010, (2): 235-236.

**263.** 唐宏亮, 庞军, 高丽芳, 等.针刺推拿治疗亚健康临床研究的系统评价[J]. 中国针灸. 2010, (8): 699-704.

**264.** 唐宏亮, 庞军, 雷龙鸣, 等.针灸治疗亚健康临床研究的系统评价[J]. 辽宁中医杂志. 2010, 37(7): 1188-1190.

**265.** 汪海飚, 罗小军.针刺治疗呃逆疗效的系统评价[J]. 中医临床研究. 2011, (17): 50-52.

**266.** 王春阳, 韩瑞发.针灸治疗慢性前列腺炎的 Meta 分析[J]. 中华男科学杂志. 2008, 14(9): 853-856.

**267.** 王德华, 罗永岚, 李畅.艾灸治疗溃疡性结肠炎疗效的Meta分析[J]. 辽宁中医杂志. 2011, (11): 2247-2248.

**268.** 王凡, 张彤, 刘毅.针灸治疗坐骨神经痛的系统评价[J]. 世界中医药 ISTIC. 2007, 2(6).

**269.** 王杰, 王千怀, 武峻艳.针灸治疗原发性三叉神经痛的 Meta-分析[J]. 中华中医药杂志. 2010, 12022.

**270.** 王京京, 宋玉静, 吴中朝, 等.针灸治疗慢性疲劳综合征随机对照试验的 Meta 分析[J]. 针刺研究. 2009, (6): 421-428.

**271.** 王静华, 陈洪沛, 陈佳.火针为主治疗带状疱疹随机对照试验的系统评价[J]. 针灸临床杂志. 2009, 25(6): 16-18.

**272.** 王丽平, 解越.针灸治疗卒中后吞咽困难的系统评价[J]. 中国针灸. 2006, 26(2): 141-146.

**273.** 王珑, 迟庆滨.电针治疗抑郁症随机对照试验的系统评价[J]. 上海针灸杂志. 2008, 27(3): 36-38.

**274.** 王珑, 孙冬玮, 邹伟, 等.针刺治疗抑郁症疗效与安全性的系统评价[J]. 中国针灸. 2008, 28(5): 381-386.

**275.** 王栩, 杜元灏, 熊俊, 等.针灸与康复疗法治疗小儿脑性瘫痪疗效比较的系统评价[J]. 辽宁中医杂志. 2011, (3): 408-413.

**276.** 王妍文, 符文彬, 欧爱华, 等.腹针治疗颈椎病临床随机对照研究的系统评价[J]. 针刺研究. 2011, 36(2): 137-144.

**277.** 王妍文, 符文彬, 彭汉郭, 等.温针灸治疗颈椎病临床随机对照试验的系统评价[J]. 辽宁中医杂志. 2011, 38(2): 340-344.

**278.** 王云娜, 黎波, 杜元灏, 等.针灸治疗神经性皮炎临床随机对照试验疗效及安全性评价[J]. 辽宁中医杂志. 2009, 36(12): 2160-2163.

**279.** 王再岭, 傅立新, 熊俊, 等.针刺治疗脑卒中后尿失禁疗效的系统评价[J]. 针灸临床杂志. 2010, (1).

**280.** 魏乙锋, 高淑红.针刺与西药治疗带状疱疹后遗神经痛疗效比较的系统评价[J]. 河南中医. 2011, (11): 1324-1327.

**281.** 吴滨, 温春毅, 石健林, 等.针灸戒毒的荟萃分析[J]. 中国针灸. 2003, 23(9): 501-505.

**282.** 吴远鹏, 石兰岚, 杨拯, 等.针灸治疗粘连性肠梗阻疗效的Meta分析[J]. 中医药导报. 2011, (5): 125-128.

**283.** 肖丽, 陈元武, 杜元灏, 等.针灸治疗抽动秽语综合征临床随机对照试验系统评价[J]. 时珍国医国药. 2010, 21(5): 1199-1202.

**284.** 肖丽, 黎波, 杜元灏, 等.针灸治疗变应性鼻炎临床随机对照试验系统评价[J]. 中国针灸. 2009, (6): 512-516.

**285.** 谢宜南, 王舒, 赵然, 等.针刺治疗不安腿综合征疗效的系统评价[J]. 针灸临床杂志. 2011, (6): 7-10.

**286.** 熊俊, 陈日新, 付勇, 等.热敏灸治疗腰椎间盘突出症随机对照试验的系统评价[J]. 江西中医药. 2011, 42(3): 48-51.

**287.** 熊俊, 杜元灏, 刘佳琳, 等.针灸与西药治疗抑郁性神经症疗效比较的系统评价[J]. 中国循证医学杂志. 2009, 9(9): 969-975.

**288.** 徐海蓉, 刘志顺, 赵宏.针刺治疗膀胱过度活动症的系统评价[J]. 现代中西医结合杂志. 2011, 20(4): 393-399.

**289.** 徐旭东, 吴宇驹.电针对抑郁症治疗效果的 Meta 分析[J]. 临床精神医学杂志. 2008, 18(2): 111-112.

**290.** 许辛寅, 江钢辉.CT 定位围针刺法治疗中风疗效的系统评价[J]. 中华实用中西医杂志. 2008, 21(15): 1273-1276.

**291.** 杨芳, 王义亮, 郭强, 等.针灸治疗神经性皮炎的文献系统评价[J]. 针灸临床杂志. 2010, (8).

**292.** 杨光, 王新卷.电针治疗颈椎病疗效的系统评价[J]. 北京中医. 2006, 25(7): 433-435.

**293.** 杨丽红, 杜元灏, 熊俊, 等.1 针灸治疗帕金森病疗效的系统评价[J]. 中国循证医学杂志. 2010, 10(6): 711-717.

**294.** 杨珊莉, 陈立典, 陶静, 等.功能训练结合针刺治疗脑卒中运动功能障碍的系统评价[J]. 中国康复医学杂志. 2008, 23(7): 649-652.

**295.** 杨志波, 张文娟.针灸治疗湿疹的系统评价[J]. 国际中医中药杂志. 2010, 32(3): 250-251.

**296.** 杨志新, 石学敏.醒脑开窍针刺法治疗中风疗效与安全性的系统评价[J]. 中国针灸. 2007, 27(8): 601-608.

**297.** 移康, 王云芳, 田金徽, 等.针灸治疗类风湿性关节炎的系统评价[J]. 甘肃科技. 2008, 24(17): 144-146.

**298.** 于金娜, 刘保延, 刘志顺, 等.针刺治疗乳腺结构不良的临床疗效和安全性评价[J]. 中国循证医学杂志. 2005, 5(5): 381-403.

**299.** 于璐, 张燕, 陈程, 等.针灸治疗哮喘随机对照临床研究文献Meta分析[J]. 中国针灸. 2010, 9787-792.

**300.** 余芝, 鞠传慧, 徐斌, 等.针刺治疗单纯性肥胖的临床随机对照试验系统评价[J]. 时珍国医国药. 2010, 21(2): 434-436.

**301.** 虞先敏, 那木海, 陈跃来.针刺治疗慢性无菌性前列腺炎文献系统评价[J]. 上海中医药大学学报. 2009, 23(4): 47-49.

**302.** 虞先敏, 朱国苗, 陈跃来, 等.针刺治疗带状疱疹的国内文献系统评价[J]. 中国针灸. 2007, 27(7): 536-540.

**303.** 喻自峰.针刺辅助治疗糖尿病足疗效的Meta分析[J]. 中华内分泌外科杂志. 2011, 5(6).

**304.** 袁梦郎, 杨拯, 呙金海, 等.针刺结合康复功能训练治疗脑卒中后吞咽障碍临床疗效的Meta分析[J]. 中国康复医学杂志. 2011, (5): 467-470.

**305.** 袁思斯, 张树怡.针灸治疗脑卒中后认知障碍的 Meta 分析[J]. 中国民族民间医药杂志. 2010, 19(9): 47-48.

**306.** 岳淑娟, 傅立新, 卢引明, 等.针刺与药物治疗广泛性焦虑症疗效的系统评价[J]. 针灸临床杂志. 2009, 25(5): 42-44.

**307.** 张碧云, 牛健民, 温济英.针刺对分娩效果的 Meta 分析[J]. 山东中医杂志. 2007, 26(8): 547-549.

**308.** 张光彩, 黄叶飞, 朱晓平, 等.针灸治疗中风后抑郁症疗效的Meta分析[J]. 新中医. 2011, (2): 127-129.

**309.** 张建博, 任路, 孙艳.针刺治疗中风后抑郁症的 Meta 分析[J]. 中国针灸. 2009, (7): 599-602.

**310.** 张世洪, 刘鸣, 李林.针刺治疗急性脑卒中随机或半随机对照试验的 Cochrane 系统评价[J]. 中国临床康复. 2005, 9(9): 108-110.

**311.** 张彤, 张莉, 张惠敏, 等.针刺治疗急性缺血性卒中的系统评价[J]. 中华中医药杂志. 2009, 24(1): 101-104.

**312.** 张维, 刘志顺, 彭唯娜.针刺治疗良性前列腺增生症的系统评价[J]. 循证医学. 2006, 6(5): 291-296.

**313.** 张维, 彭唯娜, 刘志顺.针刺治疗慢性疲劳综合征的系统评价[J]. 循证医学. 2009, 9(1): 41-47.

**314.** 张议元, 熊俊, 杜元灏.针灸与西药治疗更年期综合征疗效比较的系统评价[J]. 辽宁中医杂志. 2011, (3): 538-544.

**315.** 赵琛, 穆敬平, 崔云华, 等.针灸治疗肠易激综合征的 Meta 分析[J]. 中华中医药学刊. 2010, 5(28): 96l-963.

**316.** 赵铭辉, 黄小冬, 熊俊, 等.针刺治疗变应性鼻炎疗效的系统评价[J]. 中国中西医结合耳鼻咽喉科杂志. 2009, 17(6): 309-312.

**317.** 赵强, 蔡宝君.国内针推并用治疗椎动脉型颈椎病疗效的系统评价[J]. 长春中医药大学学报. 2010, 26(2): 217-218.

**318.** 赵强, 冯伟, 蔡宝君.国内温针治疗膝关节骨陛关节炎疗效的系统评价[J]. 辽宁中医药大学学报. 2010, 12(4): 49-51.

**319.** 赵然, 傅立新, 熊俊, 等.针刺治疗原发性高血压病远期疗效的系统评价[J]. 针灸临床杂志. 2011, (3): 46-51.

**320.** 赵婷, 王映辉.针灸治疗带状疱疹随机对照试验的系统评价[J]. 上海针灸杂志. 2007, 26(6): 30-33.

**321.** 郑春爱, 徐立.艾灸治疗带状疱疹的临床随机对照试验Meta分析[J]. 针灸临床杂志. 2011, (11): 48-50.

**322.** 钟宝亮, 黄悦勤, 李会娟.针灸治疗抑郁症疗效和安全性的系统评价[J]. 中国心理卫生杂志. 2008, 22(9): 641-647.

**323.** 周璇, 王琦.穴位按压腕带缓解术后恶心呕吐随机对照试验的Meta分析[J]. 护理学杂志. 2011, (6): 81-84.

**324.** 朱丹, 吕黄伟, 李晓倩, 等.1 P6 刺激对术后恶心呕吐有效性的 Meta 分析[J]. 中国循证医学杂志. 2010, 10(8): 923-931.

**325.** 朱丽凌, 王卫星, 郭小刚.针刺治疗脑卒中后呃逆疗效与安全性的系统评价[J]. 中国循证医学杂志. 2011, (3): 325-328.

**326.** 朱蔓佳, 张虹.不同针刺方法治疗血管性痴呆的 Meta-分析[J]. 辽宁中医杂志. 2009, (009): 1475-1477.

***Updated from 2012 to 2013:***

1. Ba J, Wu Y, Li Y, et al.Updated meta-analysis of acupuncture for treating dry Eye[J]. Medical Acupuncture. 2013, 25(5): 317-327.

2. Cao H, Wang Y, Chang D, et al.Acupuncture for vascular mild cognitive impairment: a systematic review of randomised controlled trials[J]. Acupunct Med. 2013, 31(4): 368-374.

3. Cao HJ.Acupoint stimulation for acne: A systematic review of randomized controlled trials[J]. Journal of Alternative and Complementary Medicine. 2013, 19(7): A16-A17.

4. Cao L, Zhang XL, Gao YS, et al.Needle acupuncture for osteoarthritis of the knee A systematic review and updated meta-analysis[J]. Saudi Medical Journal. 2012, 33(5): 526-532.

5. Chae Y, Chang DS, Lee SH, et al.Inserting Needles Into the Body: A Meta-Analysis of Brain Activity Associated With Acupuncture Needle Stimulation[J]. Journal Of Pain. 2013, 14(3): 215-222.

6. Chen HY, Li SG, Cho WC, et al.The role of acupoint stimulation as an adjunct therapy for lung cancer: a systematic review and meta-analysis[J]. BMC Complement Altern Med. 2013, 13362.

7. Chen J, Ren Y, Tang Y, et al.Acupuncture therapy for angina pectoris: a systematic review[J]. J Tradit Chin Med. 2012, 32(4): 494-501.

8. Chen MN, Chien LW, Liu CF.Acupuncture or Acupressure at the Sanyinjiao (SP6) Acupoint for the Treatment of Primary Dysmenorrhea: A Meta-Analysis[J]. Evidence-Based Complementary And Alternative Medicine. 2013.

9. Wong V, Cheuk Daniel KL, Lee S, et al.Acupuncture for acute management and rehabilitation of traumatic brain injury[J]. Cochrane Database of Systematic Reviews. 2013, (3).

10. Chen W, Yang GY, Liu B, et al.Manual Acupuncture for Treatment of Diabetic Peripheral Neuropathy: A Systematic Review of Randomized Controlled Trials[J]. Plos One. 2013, 8(9).

11. Chen YW, Wang HH.The Effectiveness of Acupressure on Relieving Pain: A Systematic Review[J]. Pain Manag Nurs. 2013.

12. Cheong KB, Zhang JP, Huang Y, et al.The effectiveness of acupuncture in prevention and treatment of postoperative nausea and vomiting - a systematic review and meta-analysis[J]. PLoS One. 2013, 8(12): e82474.

13. Choi TY, Choi J, Kim KH, et al.Moxibustion for the treatment of osteoarthritis: a systematic review and meta-analysis[J]. Rheumatology International. 2012, 32(10): 2969-2978.

14. Choi TY, Lee MS, Ernst E.Acupuncture for cancer patients suffering from hiccups: A systematic review and meta-analysis[J]. Complementary Therapies In Medicine. 2012, 20(6): 447-455.

15. Chung YC, Chen HH, Yeh ML.Acupoint stimulation intervention for people with primary dysmenorrhea: Systematic review and meta-analysis of randomized trials[J]. Complementary Therapies In Medicine. 2012, 20(5): 353-363.

16. Clark RJ, Tighe M.The effectiveness of acupuncture for plantar heel pain: a systematic review[J]. Acupuncture In Medicine. 2012, 30(4): 298-306.

17. Franconi G, Manni L, Schroder S, et al.A Systematic Review of Experimental and Clinical Acupuncture in Chemotherapy-Induced Peripheral Neuropathy[J]. Evidence-Based Complementary And Alternative Medicine. 2013.

18. Hao CZ, Wu F, Guo Y, et al.Acupuncture for neurogenic bladder after spinal cord injury: A systematic review and meta-analysis[J]. European Journal Of Integrative Medicine. 2013, 5(2): 100-108.

19. Hao XA, Xue CC, Dong L, et al.Factors associated with conflicting findings on acupuncture for tension-type headache: qualitative and quantitative analyses[J]. J Altern Complement Med. 2013, 19(4): 285-297.

20. Lee JA, Park SW, Hwang PW, et al.Acupuncture for Shoulder Pain After Stroke: A Systematic Review[J]. Journal Of Alternative And Complementary Medicine. 2012, 18(9): 818-823.

21. He XR, Wang Q, Li PP.Acupuncture and moxibustion for cancer-related fatigue: a systematic review and meta-analysis[J]. Asian Pac J Cancer Prev. 2013, 14(5): 3067-3074.

22. Heo I, Shin BC, Kim YD, et al.Acupuncture for Spinal Cord Injury and Its Complications: A Systematic Review and Meta-Analysis of Randomized Controlled Trials[J]. Evidence-Based Complementary And Alternative Medicine. 2013.

23. Holmer Pettersson P, Wengstrom Y.Acupuncture prior to surgery to minimise postoperative nausea and vomiting: a systematic review[J]. J Clin Nurs. 2012, 21(13-14): 1799-1805.

24. Hutchinson AJ, Ball S, Andrews JC, et al.The effectiveness of acupuncture in treating chronic non-specific low back pain: a systematic review of the literature[J]. J Orthop Surg Res. 2012, 736.

25. Ji J, Lu Y, Liu H, et al.Acupuncture and moxibustion for inflammatory bowel diseases: a systematic review and meta-analysis of randomized controlled trials[J]. Evid Based Complement Alternat Med. 2013, 2013158352.

26. Jiang HR, Ni S, Li JL, et al.Systematic Review of Randomized Clinical Trials of Acupressure Therapy for Primary Dysmenorrhea[J]. Evidence-Based Complementary And Alternative Medicine. 2013.

27. Kietrys DM, Palombaro KM, Azzaretto E, et al.Effectiveness of Dry Needling for Upper-Quarter Myofascial Pain: A Systematic Review and Meta-analysis[J]. Journal Of Orthopaedic & Sports Physical Therapy. 2013, 43(9): 620-634.

28. Kim JI, Choi JY, Lee DH, et al.Acupuncture for the treatment of tinnitus: a systematic review of randomized clinical trials[J]. Bmc Complementary And Alternative Medicine. 2012, 12.

29. Kim JI, Lee MS, Choi TY, et al.Acupuncture for Bell's Palsy: A Systematic Review and Meta-analysis[J]. Chinese Journal Of Integrative Medicine. 2012, 18(1): 48-55.

30. Kim K, Noh S, Lee B, et al.Acupuncture for lumbar spinal stenosis: A systematic review[J]. BMC Complementary and Alternative Medicine. 2012, 12.

31. Kim KH, Kim TH, Lee BR, et al.Acupuncture for lumbar spinal stenosis: A systematic review and meta-analysis[J]. Complementary Therapies In Medicine. 2013, 21(5): 535-556.

32. Kim KH, Lee BR, Ryu JH, et al.The role of acupuncture in emergency department settings: A systematic review[J]. Complementary Therapies In Medicine. 2013, 21(1): 65-72.

33. Kim YC, Lee MS, Park ES, et al.Acupressure for the Treatment of Musculoskeletal Pain Conditions: A Systematic Review[J]. Journal Of Musculoskeletal Pain. 2012, 20(2): 116-121.

34. Kim YD, Heo I, Shin BC, et al.Acupuncture for Posttraumatic Stress Disorder: A Systematic Review of Randomized Controlled Trials and Prospective Clinical Trials[J]. Evidence-Based Complementary And Alternative Medicine. 2013.

35. Lam M, Galvin R, Curry P.Effectiveness of acupuncture for nonspecific chronic low back pain: a systematic review and meta-analysis[J]. Spine (Phila Pa 1976). 2013, 38(24): 2124-2138.

36. Lee C, Crawford C, Wallerstedt D, et al.The effectiveness of acupuncture research across components of the trauma spectrum response (tsr): a systematic review of reviews[J]. Syst Rev. 2012, 146.

37. Lee H, Lee JH, Choi TY, et al.Acupuncture for acute low back pain: A systematic review[J]. Clinical Journal of Pain. 2013, 29(2): 172-185.

38. Lee HS, Park HL, Lee SJ, et al.Scalp acupuncture for Parkinson's disease: A systematic review of randomized controlled trials[J]. Chinese Journal Of Integrative Medicine. 2013, 19(4): 297-306.

39. Park JW, Lee BH, Lee H.Moxibustion in the management of irritable bowel syndrome: systematic review and meta-analysis[J]. Bmc Complementary And Alternative Medicine. 2013, 13.

40. Park J, Hahn S, Park JY, et al.Acupuncture for ankle sprain: systematic review and meta-analysis[J]. Bmc Complementary And Alternative Medicine. 2013, 13.

41. Lee SJ, Shin BC, Lee MS, et al.Scalp acupuncture for stroke recovery: A systematic review and meta-analysis of randomized controlled trials[J]. European Journal Of Integrative Medicine. 2013, 5(2): 87-99.

42. Lee WB, Woo SH, Min BI, et al.Acupuncture for gouty arthritis: a concise report of a systematic and meta-analysis approach[J]. Rheumatology. 2013, 52(7): 1225-1232.

43. Leung MCP, Yip KK, Lam CT, et al.Acupuncture improves cognitive function A systematic review star(Delta circle)[J]. Neural Regeneration Research. 2013, 8(18): 1673-1684.

44. Li F, Gao Z, Jing J, et al.Effect of point application on chronic obstructive pulmonary disease in stationary phase and effects on pulmonary function: a systematic evaluation of randomized controlled trials[J]. J Tradit Chin Med. 2012, 32(4): 502-514.

45. Lian WL, Pan MQ, Zhou DH, et al.Effectiveness of acupuncture for palliative care in cancer patients: A systematic review[J]. Chin J Integr Med. 2013.

46. Long YB, Wu XP.A meta-analysis of the efficacy of acupuncture in treating dysphagia in patients with a stroke[J]. Acupuncture In Medicine. 2012, 30(4): 291-297.

47. MacPherson H, Corbett M, Rice S, et al.Acupuncture and other physical treatments for the relief of chronic pain due to osteoarthritis of the knee: Network meta-analysis[J]. Journal of Alternative and Complementary Medicine. 2013, 19(7): A2.

48. Manheimer E, van der Windt D, Cheng K, et al.The effects of acupuncture on rates of clinical pregnancy among women undergoing in vitro fertilization: a systematic review and meta-analysis[J]. Hum Reprod Update. 2013, 19(6): 696-713.

49. Mannix SM, O'Sullivan C, Kelly GA.Acupuncture for managing phantom-limb syndrome: A systematic review[J]. Medical Acupuncture. 2013, 25(1): 23-42.

50. Yeung WF, Chung KF, Poon MM, et al.Acupressure, reflexology, and auricular acupressure for insomnia: a systematic review of randomized controlled trials[J]. Sleep Med. 2012, 13(8): 971-984.

51. Zeng Y, Luo T, Finnegan-John J, et al.Meta-Analysis of Randomized Controlled Trials of Acupuncture for Cancer-Related Fatigue[J]. Integr Cancer Ther. 2013.

52 Zhang CS, Yang AW, Zhang AL, et al.Sham Control Methods Used in Ear-Acupuncture/Ear-Acupressure Randomized Controlled Trials: A Systematic Review[J]. J Altern Complement Med. 2013.

53. Posadzki P, Moon TW, Choi TY, et al.Acupuncture for cancer-related fatigue: a systematic review of randomized clinical trials[J]. Supportive Care In Cancer. 2013, 21(7): 2067-2073.

54. Posadzki P, Zhang J, Lee MS, et al.Acupuncture for chronic nonbacterial prostatitis/chronic pelvic pain syndrome: a systematic review[J]. J Androl. 2012, 33(1): 15-21.

55. Qu F, Zhou J, Ren RX.Effects of Acupuncture on the Outcomes of In Vitro Fertilization: A Systematic Review and Meta-Analysis[J]. Journal Of Alternative And Complementary Medicine. 2012, 18(5): 429-439.

56. Raith W, Urlesberger B, Schmolzer GM.Efficacy and Safety of Acupuncture in Preterm and Term Infants[J]. Evidence-Based Complementary And Alternative Medicine. 2013.

57. Rixin Chen JX, Zhenhai Chi,Bo Zhang,Acupuncture, Rehabilitation Department TAHoJUoTCM, Nanchang 330006,China, Key Laboratory of Heat-sensitive Moxibustion SAoTCMoPsRoC, Nanchang 330006,China, et al.Heat-sensitive moxibustion for lumbar disc herniation:a meta-analysis of randomized controlled trials[J]. Journal of Traditional Chinese Medicine. 2012, (03).

58. Selva Olid A, Martinez Zapata MJ, Sola I, et al.Efficacy and safety of needle acupuncture for treating gynecologic and obstetric disorders: An overview[J]. Medical Acupuncture. 2013, 25(6): 386-397.

59. Sui Y, Zhao HL, Wong VC, et al.A systematic review on use of Chinese medicine and acupuncture for treatment of obesity[J]. Obes Rev. 2012, 13(5): 409-430.

60. Tahiri M, Mottillo S, Joseph L, et al.Alternative Smoking Cessation Aids: A Meta-analysis of Randomized Controlled Trials[J]. American Journal Of Medicine. 2012, 125(6): 576-584.

61. Urroz P, Colagiuri B, Smith CA, et al.Effect of Acute Acupuncture Treatment on Exercise Performance and Postexercise Recovery: A Systematic Review[J]. Journal Of Alternative And Complementary Medicine. 2013, 19(1): 9-16.

62. Wang J, Xiong X, Liu W.Acupuncture for essential hypertension[J]. Int J Cardiol. 2013, 169(5): 317-326.

63. Wang QP, Bai M, Lei D.Effectiveness of Acupuncture in Treatment of Facial Spasm: A Meta-analysis[J]. Alternative Therapies In Health And Medicine. 2012, 18(3): 45-52.

64. Wang Y, Shen J, Wang XM, et al.Scalp acupuncture for acute ischemic stroke: A meta-analysis of randomized controlled trials[J]. Evidence-based Complementary and Alternative Medicine. 2012, 2012.

65. Wong ISY, Ng KF, Tsang HWH.Acupuncture for dysphagia following stroke: A systematic review[J]. European Journal Of Integrative Medicine. 2012, 4(2): E141-E150.

66. Xiong X, Liu W, Yang X, et al.Moxibustion for essential hypertension[J]. Complementary Therapies in Medicine. 2013.

67. Xu M, Yan S, Yin X, et al.Acupuncture for chronic low back pain in long-term follow-up: a meta-analysis of 13 randomized controlled trials[J]. Am J Chin Med. 2013, 41(1): 1-19.

68. Yan X, Zhu T, Ma C, et al.A meta-analysis of randomized controlled trials on acupuncture for amblyopia[J]. Evid Based Complement Alternat Med. 2013, 2013648054.

69. Yang M, Li X, Liu S, et al.Meta-analysis of acupuncture for relieving non-organic dyspeptic symptoms suggestive of diabetic gastroparesis[J]. BMC Complement Altern Med. 2013, 13(1): 311.

70. Yeung WF, Chung KF, Poon MM, et al.Prescription of chinese herbal medicine and selection of acupoints in pattern-based traditional chinese medicine treatment for insomnia: a systematic review[J]. Evid Based Complement Alternat Med. 2012, 2012902578.

71. Zheng CH, Zhang MM, Huang GY, et al.The Role of Acupuncture in Assisted Reproductive Technology[J]. Evidence-Based Complementary And Alternative Medicine. 2012.

72. Zhuang L, Yang Z, Zeng X, et al.The preventive and therapeutic effect of acupuncture for radiation-induced xerostomia in patients with head and neck cancer: a systematic review[J]. Integr Cancer Ther. 2013, 12(3): 197-205.

73. ZHANG GC, FU WB, XU NG, et al. Meta analysis of the curative effect of acupuncture on post-stroke depression[J]. Journal of Traditional Chinese Medicine. 2012, (01).

74. ZHOU JW, LI J, ZHAO JJ, et al. Scalp acupuncture for stroke recovery: A systematic review and meta-analysis of randomized controlled trials [J]. World Journal of Acupuncture-Moxibustion. 2013, (02).

75. Zhang J, Li X, Xu J, et al.Laser acupuncture for the treatment of asthma in children: a systematic review of randomized controlled trials[J]. J Asthma. 2012, 49(7): 773-777.

76. Zhang QH, Yue JH, Liu M, et al.Moxibustion for the Correction of Nonvertex Presentation: A Systematic Review and Meta-Analysis of Randomized Controlled Trials[J]. Evidence-Based Complementary And Alternative Medicine. 2013.

77. Zhang T, Chon TY, Liu B, et al.Efficacy of acupuncture for chronic constipation: a systematic review[J]. Am J Chin Med. 2013, 41(4): 717-742.

78. Zhao XF, Du Y, Liu PG, et al.Acupuncture for Stroke: Evidence of Effectiveness, Safety, and Cost From Systematic Reviews[J]. Topics In Stroke Rehabilitation. 2012, 19(3): 226-233.

79. Zheng CH.Effects of acupuncture on pregnancy rates in women undergoing in vitro fertilization: a systematic review and meta-analysis (vol 97, pg 599, 2012)[J]. Fertility And Sterility. 2012, 97(4): 1017-1017.

80. Cheong Ying C, Dix S, Hung Yu Ng E, et al.Acupuncture and assisted reproductive technology[J]. Cochrane Database of Systematic Reviews. 2013, (7).

81. He J, Zheng M, Zhang M, et al.Acupuncture for mumps in children[J]. Cochrane Database of Systematic Reviews. 2012, (9).

82. Kwan I, Bhattacharya S, Knox F, et al.Pain relief for women undergoing oocyte retrieval for assisted reproduction[J]. Cochrane Database of Systematic Reviews. 2013, (1).

83. Manheimer E, Cheng K, Wieland LS, et al.Acupuncture for treatment of irritable bowel syndrome[J]. Cochrane Database of Systematic Reviews. 2012, (5).

84. Wong V, Cheuk Daniel KL, Chu V.Acupuncture for hypoxic ischemic encephalopathy in neonates[J]. Cochrane Database of Systematic Reviews. 2013, (1).

85. 朱原, 傅立新, 李胜, et al.脑卒中后假性球麻痹致吞咽障碍针灸治疗的系统评价[J]. 山东中医杂志. 2012, (10).

86. 左小宏, 傅立新, 申昕, et al.针刺治疗中风后尿潴留的系统评价[J]. 针灸临床杂志, 2012, 28(11): 51-53.

87. 陆柳如,凌沛. 针药并用治疗排卵障碍性不孕的Meta分析. 内蒙古中医药, 2013 (4):33-34

88. 郭炜, 裴强伟, 董文亮, 刘西建, 孙志翠, 韩涛. 针药结合治疗原发性高血压文献的系统评价. 山东中医药大学学报, 2013,37(2):99-100,103

89. 黄予巍, 方晓丽, 严兴科. 子午流注针法治疗脑卒中随机对照试验文献的荟萃分析. 辽宁中医药大学学报, 2013,15(2):109-112

90. 李林, 袁坤, 张立恒, 王华. 电针夹脊穴治疗腰椎间盘突出症临床随机对照试验系统评价, 中国中医骨伤科杂志,2012,20(12):13-17.

91 曹巧珍 杜.针灸治疗排卵障碍性不孕症临床疗效的Meta分析[J]. 当代医学. 2012.

92. 陈璐, 李素荷, 曾侠一.针刺治疗急性期贝尔麻痹有效性与安全性的系统评价[J]. 中医杂志. 2012, (22).

93. 崔海福, 陈程, 于璐, 等.针灸治疗功能性腹泻随机对照临床研究文献的Meta分析[J]. 中华中医药杂志. 2012, (11).

94. 符文彬 聂.针灸与西药治疗脑卒中后抑郁症疗效比较的系统评价[J]. 世界中医药. 2012.

95. 付蕾, 柴华.针灸治疗带状疱疹的疗效分析[J]. 中医药临床杂志. 2012, 24(7): 619-622.

96. 韩正军, 任超展, 杜小正.针灸治疗带状疱疹急性期随机对照临床试验的Meta分析[J]. 中医研究. 2013, 26(2): 56-59.

97. 贺亚楠, 陈益清, 顾英杰, 等.脑卒中后顽固性呃逆针刺疗法康复效果的系统评价[J]. 2013, 27(34): 3959-3961.

98. 黄厚斌 戴刘张魏.针灸治疗视神经萎缩随机对照临床试验的Meta分析[J]. 中南大学学报：医学版. 2013.

99. 黎小慧, 陈俊琦, 胡亚南, 等.针药结合与西药对脑卒中后抑郁症患者抑郁状态改善情况比较的荟萃分析[J]. 陕西中医. 2012, 33(9): 1263-1267.

100. 李国义 龚吴曾李秦.国内针刺治疗耳鸣疗效的系统评价[J]. 现代中西医结合杂志. 2012.

101. 李天佐 孙王.针灸在慢性头痛治疗中的应用――临床荟萃分析[J]. 首都医科大学学报. 2012.

102. 刘安国, 严兴科, 阚丽丽, 等.铺灸疗法为主治疗强直性脊柱炎的Meta分析[J]. 西部中医药. 2013, (12): 51-54.

103. 刘福水, 郭长青, 金晓飞.针灸治疗轻、中度原发性高血压随机对照试验的Meta分析[J]. 中国中医基础医学杂志. 2012, (04).

104. 刘福水, 金晓飞, 郭长青.针灸与针刀治疗肩关节周围炎疗效比较的系统评价[J]. 中华中医药杂志. 2012, (03).

105. 刘福水, 张义, 钟鼎文, 等. 针刀与针灸治疗颈椎病疗效比较的Meta分析[J]. 中国组织工程研究. 2012, (09).

106. 刘敏, 刘迈兰, 于美玲, 等.针灸治疗干眼的系统评价[J]. Acupuncture therapy for dry eye: a systematic review. 2012, 22(4): 242-246.

107. 龙小娜 胡储孙肖宋.针刺治疗神经根型颈椎病系统评价[J]. 安徽中医学院学报. 2012.

108. 卢敏, 谭旭仪, 黄璐.针灸治疗膝关节骨性关节炎的Meta分析[J]. 中医药导报. 2012, 18(1): 81-83.

109. 马铁明.针灸疗法治疗肩手综合征疗效的Meta分析[J]. 针刺研究. 2012.

110. 马占强, 葛明.针灸治疗高脂血症安全性和有效性的荟萃分析[J]. 河南中医. 2012, 32(10): 1398-1401.

111. 潘东.针灸治疗脑卒中后失语症临床疗效的Meta分析[J]. 山东医药. 2013, 53(42): 87-89.

112. 彭川, 承欧梅.针刺治疗血管性痴呆的meta-分析[J]. 医药前沿. 2013, (17): 7-8.

113. 钱玉新.针刺之治疗原发性高血压的疗效和安全性的系统评价[J]. 北方药学. 2013, (3): 72-73.

114. 邱玲 阚王.舌针治疗中风失语症疗效的系统评价[J]. 针灸临床杂志. 2013.

115. 孙其喆, 袁梅.针灸联合穴位注射治疗肝癌癌痛的系统评价[J]. 大家健康（中旬版）. 2013, 7(6): 184-186.

116. 唐雄 吴李.针刺结合康复训练治疗脑卒中后运动功能障碍临床疗效的Meta分析[J]. 中国中医急症. 2013.

117. 唐勇 刘刘于兰.针灸治疗麻痹性斜视系统评价[J]. 辽宁中医药大学学报. 2012.

118. 田涛涛, 张玉莲, 崔远武, et al.针灸对照西药治疗老年性痴呆疗效的系统评价[J]. 长春中医药大学学报. 2012, 28(1): 48-50.

119. 田维珍 陈俞刘黄.国内针灸治疗原发性痛经的系统评价[J]. 中华中医药学刊. 2013.

120. 王富春 杜于严.针灸治疗便秘随机对照临床研究文献Meta分析[J]. 中国针灸. 2012.

121. 王强平.针灸治疗早泄疗效的Meta分析[J]. 西部医学. 2012.

122. 王巧凡, 李吉庆, 张朕华.针刺治疗原发性骨质疏松的临床荟萃分析[J]. 中国老年学杂志. 2013, 33(13): 3075-3077.

123. 王升旭 黎陈王陈.电针与抗抑郁药治疗中风后抑郁症比较的系统评价[J]. 中国全科医学. 2012.

124. 王舒 张康杨班栗岳张.针刺治疗原发性高血压的疗效及穴位频次分析[J]. 辽宁中医杂志. 2013.

125. 王文菁, 刘婕, 杨宾, 等.针药联合对冠心病治疗疗效与安全性的Meta分析[J]. 上海针灸杂志. 2012, 31(3): 202-206.

126. 王晓培 李张刘张.针灸治疗变应性鼻炎系统评价[J]. 辽宁中医药大学学报. 2013.

127. 魏素珍, 郑访江, 等.中医针药并用治疗小儿遗尿的Meta分析[J]. 中国优生优育，2013, 19(2): 78-81.

128. 吴大嵘 陈黄贡.针刺治疗术后肠梗阻相关文献的系统评价[J]. 陕西中医. 2012.

129. 吴翔 刘金.针刀与针灸治疗膝骨关节炎疗效比较的Meta分析[J]. 中国组织工程研究. 2012.

130. 吴晓亮 裴张孙耿.针灸治疗肠易激综合征Meta分析[J]. 中国针灸. 2012.

131. 吴新贵, 李趣红, 何源浩, et al.腰椎间盘突出症针灸治疗效果的系统评价[J]. 广西医科大学学报. 2013, 30(4): 562-566.

132. 徐琰, 李万瑶, 刘洁, 等. 针灸与康复治疗脑卒中后肩手综合征疗效比较的系统评价与Meta分析[J]. 时珍国医国药, 2013, 24(7): 1794-1798.

133. 严兴科, 崔海福, 陈程, et al.针灸治疗慢性前列腺炎随机对照临床研究文献的Meta分析[J]. 时珍国医国药. 2012, (10).

134. 严兴科 董刘王.针灸治疗急性胃炎随机对照临床试验的Meta分析[J]. 甘肃中医学院学报. 2013.

135. 杨蕾, 符文彬, 张光彩, 等.腹针治疗颈椎病有效性的系统评价[J]. 中华中医药杂志. 2012, (02).

136. 杨志新 董.醒脑开窍针刺法治疗中风的系统评价[J]. 中国针灸. 2013.

137. 于慧, 韩晶, 谭奇纹, et al.针刺治疗原发性高血压病的疗效分析[J]. 2013, 29(2): 39-45.

138. 余利忠, 孙作乾, 李向军, et al.针灸治疗腰椎间盘突出症随机对照临床研究文献的Meta分析[J]. 中国中医药信息杂志. 2012, (05).

139. 袁梅 孙.针灸配合均衡膳食治疗单纯性肥胖症的系统评价[J]. 医学美学美容：中旬刊. 2013.

140. 臧亮 杨高.针灸治疗心绞痛的疗效评价[J]. 针灸临床杂志. 2012.

141. 曾永保, 梅志刚, 董铮, et al.针灸治疗糖尿病膀胱临床研究的Meta分析[J]. 时珍国医国药. 2012, 23(1): 198-201.

142. 张虹 陈谢樊梁.电针治疗带状疱疹随机对照试验系统评价[J]. 河北中医. 2013.

143. 张剑锋（指导） 王邓.针刺治疗面肌痉挛随机对照临床试验Meta分析[J]. 西部中医药. 2013.

144. 张宁, 胡静, 王雁.针灸治疗围绝经期睡眠障碍随机对照研究Meta评价[J]. 中国中医药信息杂志. 2012, (08).

145. 张雄 孙.针灸治疗帕金森病非运动症状疗效的Meta分析[J]. 上海中医药大学学报. 2013.

146. 赵宏 聂刘.针灸治疗中风后肩痛系统评价[J]. 中国中医药信息杂志. 2012.

147. 针刺治疗广泛性焦虑临床研究文献的系统评.针刺治疗广泛性焦虑临床研究文献的系统评价[J]. 中国老年学杂志. 2012, 32(15): 3206-3207.

148. 郑春爱 徐.灸治痛风性关节炎的临床随机对照试验Meta分析[J]. 针灸临床杂志. 2012.

149. 郑淑美, 崔海.针刺治疗偏头痛的Meta分析[J]. 针刺治疗偏头痛的Meta分析. 2012, (06).

150. 朱静, 张立勇, 邵湘宁, et al.针灸治疗小儿脑瘫的Meta分析[J].. 2013, (11): 62-65.
